# Inside the Battle Against Acute Myeloid Leukemia: Biology, Breakthroughs, and Hope

**DOI:** 10.3390/cells15040338

**Published:** 2026-02-13

**Authors:** Jiayang Bao, Oliver Freund, Logan Sund, Wei Du

**Affiliations:** 1 Division of Hematology and Oncology, School of Medicine, University of Pittsburgh, Pittsburgh, PA 15232, USA; jib125@pitt.edu (J.B.); ojf7@pitt.edu (O.F.);; 2 UPMC Hillman Cancer Center, Pittsburgh, PA 15213, USA

**Keywords:** acute myeloid leukemia, hematopoiesis, leukemogenesis, hematopoietic stem cells, leukemia stem cells, multi-omics profiling

## Abstract

Acute myeloid leukemia (AML) is a biologically heterogeneous and clinically aggressive hematologic malignancy defined by the clonal expansion of immature myeloid progenitors, resulting in progressive bone marrow (BM) failure, peripheral cytopenias, and fatal infectious or hemorrhagic sequelae. The adverse clinical outcomes associated with AML arise from the combined effects of disrupted physiological hematopoiesis, persistence of therapy-refractory leukemic stem cells (LSCs), and extensive inter- and intratumoral genetic and epigenetic heterogeneity that underlies rapid disease progression and relapse. AML constitutes a prototypical disorder of hematopoietic dysregulation, wherein aberrant self-renewal capacity and arrested differentiation programs drive malignant transformation through the integrated influence of recurrent genomic lesions, epigenetic reprogramming, metabolic alterations, dysregulated signaling cascades, and reciprocal interactions with the BM microenvironment. These processes collectively reconfigure transcriptional landscapes and cellular hierarchies within the leukemic compartment. The objectives of this review are to provide an integrated framework for understanding AML pathobiology encompassing chromosomal abnormalities, transcriptional and epigenetic regulatory networks, and microenvironmental cues and to emphasize emerging analytical paradigms, including integrative multi-omics, single-cell and spatial technologies, and system-level approaches, which are reshaping conceptual models of malignant hematopoiesis and accelerating the development of mechanism-based therapeutic strategies.

## 1. Introduction

Acute myeloid leukemia (AML) is an aggressive hematologic malignancy characterized by clonal expansion of myeloid progenitor cells with impaired differentiation and aberrant self-renewal capacity [1,2,3,4,5]. Clinically and biologically, AML exhibits remarkable heterogeneity, encompassing diverse genetic alterations, epigenetic states, cellular hierarchies, and altered interactions with the bone marrow (BM) microenvironment [6,7]. Recent studies using single-cell and multi-omics technologies have revealed that this heterogeneity is highly dynamic, shaped by clonal evolution and context-dependent cell state transition rather than traditional lineage hierarchies. This heterogeneity underlies the variable disease course and therapeutic response observed among patients and highlights the complexity of the molecular programs governing malignant myelopoiesis [4,8].

This review follows the progression from normal hematopoiesis to malignant transformation in AML. It begins with the key cellular and molecular processes underlying normal hematopoiesis, then examines the cellular origins of AML and the genetic and epigenetic changes that drive leukemogenesis, integrating recent insights into clonal architecture, mutational order, and epigenetic reprogramming. Subsequent sections explore altered signaling pathways, leukemic stem cell (LSC) biology, intratumoral heterogeneity, and the role of the BM microenvironment in disease progression. Finally, it highlights emerging technologies that are reshaping AML research, including multi-omics approaches, single-cell and spatial analyses, and computational modeling. These methodologies have enabled system-level dissection of AML heterogeneity, reconstruction of cellular hierarchies, and identification of context-dependent vulnerabilities. Together, this framework offers an integrated, mechanism-focused view of AML and provides a foundation for future therapeutic advances.

This review was conducted based on a comprehensive literature search of the PubMed database. Relevant articles published mainly between 2010 and 2026 were retrieved using combinations of keywords such as “acute myeloid leukemia”, “hematopoiesis”, and “leukemia stem cells”. In our selection process, priority was given to seminal works, highly cited articles, and studies published in authoritative journals to ensure the reliability and relevance of the data; conference abstracts and non-original research were excluded. The reference lists of selected articles were also manually screened to identify additional relevant studies.

## 2. Normal Hematopoiesis: A Cellular and Molecular Overview

**Figure 1:**
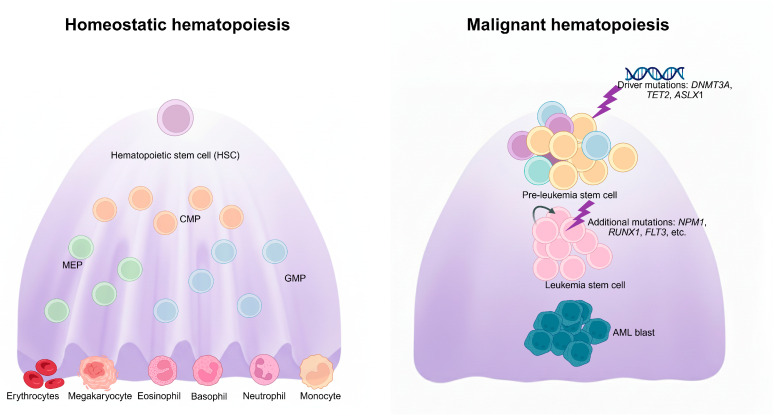
Schematic of homeostatic and malignant hematopoiesis. Under normal conditions (**left**), HSCs differentiate through intermediate progenitors, including CMP, MEP, and GMP, giving rise to mature myeloid and lymphoid lineages such as erythrocytes, megakaryocytes, eosinophils, basophils, neutrophils, and monocytes. In leukemogenesis (**right**), early driver mutations (e.g., *DNMT3A*, *TET2*, *ASXL1*) promote the emergence of pre-leukemic stem cells, which acquire additional mutations (e.g., *NPM1*, *RUNX1*, *FLT3*) to form leukemia stem cells, ultimately leading to the expansion of acute myeloid leukemia (AML) blasts. Created in BioRender. Yang, J. (2026) https://BioRender.com/hh0kzwt (accessed on 22 December 2025).

Hematopoiesis is hierarchically organized, with multipotent stem and progenitor cells giving rise to distinct myeloid and lymphoid lineages. At the apex of this hierarchy are hematopoiesis stem cells (HSCs), characterized by their capacity to self-renew and differentiate into multiple lineages (Figure 1, left panel). Recent single-cell analyses have challenged the classical stepwise model of hematopoietic differentiation, instead supporting a continuum model in which fate commitment arises gradually through intermediate transcriptional states [9,10,11,12].

HSC differentiation is tightly regulated by a complex network of transcription factors, epigenetic regulators and signals from the BM niche. In HSCs, a core set of seven transcription factors, namely FLI1, ERG, GATA2, RUNX1, TAL1, LYL1, and LMO2, known as the “heptad”, co-occupies the genome to form a highly coordinated and tightly interconnected regulatory network [13,14]. Integrated multi-omics approaches combining ChIP-seq, Hi-C, HiChIP, and histone modification profiling have shown that at the HSC–multipotent progenitor (MPP) stage, these factors extensively bind thousands of promoters and distal enhancers, establishing an open and reprogrammable regulatory landscape that maintains multipotency.

As hematopoietic stem and progenitor cells (HSPCs) undergo lineage specification, heptad binding undergoes pronounced lineage-specific remodeling. Specifically, TAL1 and GATA2, together with LYL1 and LMO2, show enhanced occupancy in megakaryocyte–erythroid regulatory regions, whereas FLI1, ERG, and RUNX1 are more active in myeloid-specific regions [13]. These shifts reflect a system-wide reorganization of transcription factor assemblies, regulatory elements, and chromatin architecture during lineage commitment [15]. Enhancers initially reinforced by the heptad are later taken over by lineage-defining transcription factors, such as PU.1 in myeloid differentiation and GATA1 in erythroid differentiation [13,16,17]. Together, these observations support a three-layer model of transcriptional control during hematopoietic lineage specification: an initial priming phase, in which the heptad establishes a multipotent regulatory framework; a remodeling phase, characterized by lineage-specific enhancer–promoter reconfiguration; and a final activation phase driven by lineage-specific transcription factors [13,15].

Epigenetic regulation further modulates lineage bias in HSCs. For example, loss of DNA methyltransferases DMNT1 or DMNT3A induces hypomethylation, skewing differentiation toward the erythroid lineage [18,19], while loss of the ten-eleven translation enzymes (TET2) favors myelomonocytic differentiation and reduces erythroid and common lymphoid progenitors [20,21]. Beyond intrinsic regulatory, HSC maintenance and fate decision are influenced by the bone marrow (BM) niche, a complex microenvironment comprising hematopoietic and mesenchymal cells, including endothelial cells, mesenchymal stem cells (MSCs), macrophages, and megakaryocytes, which provides structural support, chemokines, growth factors, and metabolic cues that regulate HSC quiescence, self-renewal, proliferation and differentiation [22,23,24,25,26].

Notably, a 2024 study integrated scRNA-seq with spatial proteomics (CODEX multiplex imaging) and generated a high-resolution spatial map of human BM, identifying a hypoxic artero-endosteal region enriched for early myeloid progenitors while peri-adipocytic zones preferentially harbored HSPCs. This finding highlights a strong correlation between the microenvironment architecture and lineage outcomes [27].

## 3. Cellular Origin of AML

AML is an aggressive hematologic malignancy originating from hematopoietic stem and progenitor cells (HSPCs) that acquire somatic mutations, ultimately conferring a proliferative advantage and clonal expansion. The process by which HSPCs accumulate somatic mutations and expand clonally is referred to as clonal hematopoiesis (CH) [28]. Over the past decade, advances in next-generation sequencing (NGS) technologies, including whole-exome sequencing (WES), whole-genome sequencing (WGS), and single-cell sequencing, have enabled high-resolution characterization of clonal architecture in clonal hematopoiesis [29,30,31]. The most common mutations are epigenetic regulators, including *DNMT3A*, *TET2* and *ASXL1*, which are considered driver mutations and account for approximately 80~90% of CH-associated variants.

CH encompasses diverse subtypes. A clinically significant subset, termed clonal hematopoiesis of indeterminate potential (CHIP), is defined by the presence of somatic mutations in leukemia-associated genes at a variant allele fraction (VAF) of ≥2% in individuals without overt hematologic malignancy or cytopenia [28,32,33]. HSCs harboring CHIP-associated mutations may be considered as pre-leukemia stem cells (pre-LSCs), particularly when they acquire additional leukemia-specific mutations that drive progression to AML [34,35,36]. Accordingly, leukemogenesis can be conceptualized as a two-stage process: initiation in pre-LSCs and transformation into full leukemia stem cells (LSCs).

Mutations in epigenetic regulator genes, such as *DNMT3A*, *TET2* and *ASXL1*, collectively referred to as “DTA” mutations, are frequently enriched in pre-LSCs, placing these cells in a transcriptionally and functionally “primed” state. Acquisition of additional mutations in genes involved in DNA damage response (DDR; e.g., *TP53*, *CHEK2*), RNA splicing (*SRSF2*, *SF3B1*, and *U2AF1*), transcriptional regulation (*RUNX1*, *GATA2*), and signaling pathway genes (*JAK2*, *KRAS*, *PTPN11*, *FLT3*) facilitates the transition from pre-LSCs to LSCs, which exhibit enhanced self-renewal, proliferative capacity, and impaired differentiation, ultimately driving AML development within the patient’s bone marrow [37,38,39] (Figure 1, right panel).

Given their central role in disease propagation, LSCs represent a critical therapeutic target in AML. A precise definition of LSCs, coupled with identification of specific biomarkers, is essential for guiding the development of targeted therapies. Finally, LSCs are distinguished from bulk leukemic blasts by their long-term self-renewal capacity and ability to reconstitute the entire leukemia hierarchy in xenotransplantation models [35,40]. Phenotypically, LSCs are heterogeneous; the classical immunophenotype is CD34^+^CD38^−^, but additional markers, including CD123, TIM3, C96, CLL-1, CD25, CD32 and CD47, have been identified to distinguish LSCs from normal HSCs. Several of these markers have been validated as functional and possess potential therapeutic relevance [41,42,43,44,45,46,47].

## 4. Genetic Mechanisms of Leukemogenesis

Building on the framework of pre-LSCs and LSCs, the pathogenesis of AML is now recognized as a multilayered process involving coordinated alterations in genetic, epigenetic, splicing, transcriptional, and high-order chromatin regulatory programs. This expanded understanding has driven systemic efforts to classify AML-associated mutations, enabling improved disease stratification and informing diagnostic and therapeutic guidelines.

Early models of AML leukemogenesis were largely based on the “two-hit” hypothesis, in which mutations were broadly categorized into class I or class II. Class I mutations, such as activating alterations in *FLT3* or *RAS*, primarily confer proliferative and survival advantages, resulting in hyperproliferation but remaining insufficient for complete leukemic transformation. In contrast, class II mutations, typically involving transcription factors or fusion oncogenes such as *PML/RARα*, *AML1/ETO*, *CEPBA*, *RUNX1-RUNX1T1*, and *CBFB-MYH11*, impair hematopoietic differentiation and cooperate with class I mutations to induce overt AML [48,49].

**Figure 2:**
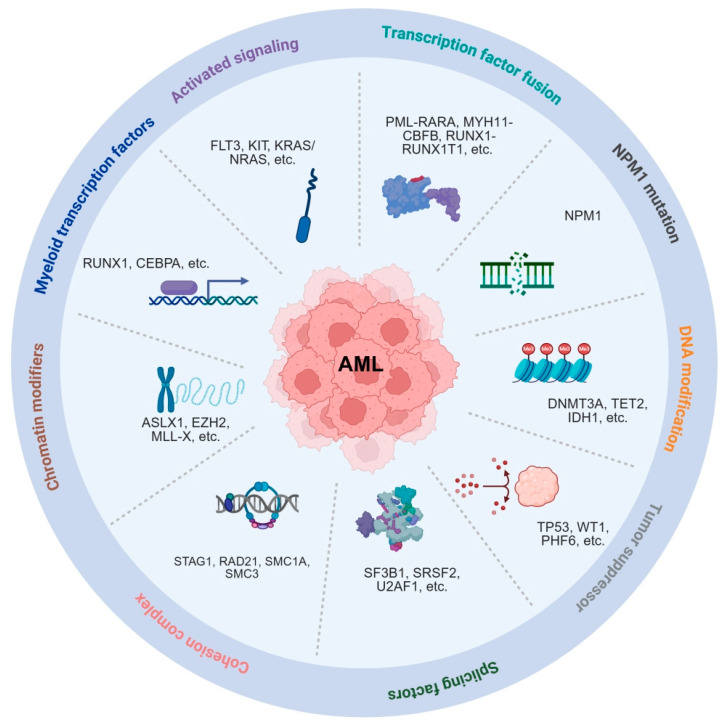
Summary of recurrent mutations and their pathways. Mutations are grouped into functional categories, including transcription factor fusions (*PML-RARA*, *MYH11-CBFB*, *RUNX1-RUNX1T1*); *NPM1* mutations; tumor suppressor genes (*TP53*, *WT1*, *PHF6*, etc.); DNA methylation regulators (*DNMT3A*, *TET2*, *IDH1*, etc.); activated signaling pathways (*FLT3*, *KIT*, *KRAS*, *NRAS*, etc.); myeloid transcription factors (*RUNX1*, *CEBPA*, etc.); chromatin modifiers (*MLL-X* fusions, *ASXL1*, *EZH2*, etc.); cohesion genes (*STAG1*, *RAD21*, *SMC1A*, *SMC3*); and spliceosome genes (*SF3B1*, *SRSF2*, *U2AF1*, etc.). Created in BioRender. Yang, J. (2026) https://BioRender.com/hh0kzwt (accessed on 22 December 2025).

In 2013, a landmark study published in *The New England Journal of Medicine (NEJM)* performed comprehensive genomic profiling of 200 paired de novo adult AML samples and categorized recurrent drive mutations into nine functional classes (Table 1). These include transcription factor fusions (*PML-RARA*, *MYH11-CBFB*, *RUNX1-RUNX1T1*, *PICALM-MLLT10*); nucleophosmin (*NPM1*) mutations; tumor suppressor genes (*TP53*, *WT1*, *PHF6*); DNA methylation regulators (*DNMT3A*, *DNMT3B*, *DNMT1*, *TET1*, *TET2*, *IDH1*, *IDH2*); activated signaling pathways (*FLT3*, *KIT*, other Tyr-kinases, Ser-Thr-kinases, *KRAS*/*NRAS*, PTPs); myeloid transcription factors (*RUNX1*, *CEBPA*, other myeloid TFs); chromatin modifiers (*MLL-X* fusions, *MLL-PTD*, *NUP98-NSD1*, *ASXL1*, *EZH2*, *KDM6A*, and other modifiers); cohesion genes; and spliceosome genes [29]. This study revealed extensive patterns of mutation co-occurrence and mutual exclusivity, highlighting distinct leukemogenic trajectories (Figure 2).

One of the most striking observations was the frequent co-occurrence of *FLT3*, *DNMT3A*, and *NPM1* mutations. AML cases harboring this triad formed a distinct molecular subgroup characterized by unique mRNA, miRNA, and DNA methylation signatures, suggesting the existence of a discrete AML subtype, a finding consistently validated in subsequent studies [29,50,51,52]. In contrast, core-binding factors (CBF) and *PML-RARA* fusion leukemias, as well as *KMT2A*/*MLL* rearrangements, were largely mutually exclusive with *NPM1* and *DNMT3A* mutations. Similarly, *RUNX1* and *TP52* mutations showed mutual exclusivity with *FLT3* and *NPM1* alterations [53,54].

These patterns of mutual exclusivity indicate that certain transcription factor fusions are individually sufficient to drive leukemogenesis. Mechanistically, *PML-RARA* arising from (t(15;17)) functions as a dominant transcriptional repressor by recruiting corepressor complexes (e.g., NCOR/SMART), disrupting PML nuclear bodies and enforcing a block in granulocytic differentiation [55,56]. The *RUNX1-RUNX1T1* fusion protein generated by (t(8;21)) recruits HDACs, DNMTs, and other corepressor complexes to *RUNX1* target loci, resulting in widespread repression of myeloid differentiation programs and maintenance of a self-renewing progenitor state [57]. The *CBFB-MYH11* fusion associated with (inv(16)) sequesters RUNX1 (the α-subunit of CBF) in cytoplasmic complexes through interactions with cytoskeletal and myosin-associated proteins, thereby abrogating nuclear core-binding factor activity and perturbing downstream differentiation pathways [54]. In *KMT2A*/*MLL*-rearranged AML, aberrant recruitment of cofactors such as Menin and DOT1L leads to ectopic H3K4 and H3K79 methylation, driving sustained activation of oncogenic transcriptional programs, including *HOXA/B*, *MEIS1* and *PBX3*, imposing a stem-cell-like chromatin state associated with aggressive, differentiation-blocked leukemia [58,59].

Collectively, these observations underscore that the mutational architecture of AML reflects functional cooperation among distinct molecular lesions. A prototypical example is the synergistic interaction between *DNMT3A* loss-of-function mutations and *FLT3-ITD* signaling. In murine models, *Dnmt3a* deficiency induces widespread DNA hypomethylation, including enhancer demethylation at key stemness-associated loci such as *MEIS1*, thereby establishing a permissive epigenetic landscape that primes HSCs but is insufficient for leukemic transformation. Subsequent acquisition of *FLT3-ITD* provides constitutive proliferative and survival signals, promoting expansion and enhanced self-renewal of pre-LSCs, and ultimately driving overt AML development [60,61,62,63]. This cooperative mechanism is strongly supported by clinical data, as patients harboring concurrent *DNMT3A* and *FLT3* mutations exhibit pronounced hypomethylation signatures and significantly inferior clinical outcomes [53,64]. Together, these findings highlight a paradigmatic model of synergetic leukemogenesis in AML, in which epigenetic instability mediated by *DNMT3A* mutations collaborates with oncogenic signaling pathways to promote malignant transformation and disease aggressiveness. Such functional cooperation explains the recurrent co-occurrence of specific mutation combinations in AML and underscores why individual lesions are rarely sufficient to induce leukemia in isolation. Understanding these cooperative genetic networks is essential for the rational design of combination therapeutic strategies.

## 5. Epigenetic Dysregulation in AML

Epigenetic dysregulation including DNA methylation, histone modification, chromatin remodeling, and noncoding RNA and RNA modifications constitutes a fundamental hallmark of AML, profoundly influencing leukemogenic mechanisms, leukemia classification and therapeutic development. Genomic studies have demonstrated that more than 60% of AML cases harbor mutations involved in epigenetic dysregulation [29,52]. These alterations strongly influence leukemic gene expression programs and contribute to disease initiation and progression, making epigenetic regulators attractive targets for AML therapy.

### 5.1. DNA Methylation and Demethylation

AML exhibits profound mutations in DNA methylation patterns. DNMT3A, a *de novo* DNA methyltransferase responsible for catalyzing 5′-cytosine methylation, is one of the most frequently mutated epigenetic regulators in AML [65]. Somatic mutations in *DNMT3A* were first reported in 2010, and are present in over 20% of AML patients, with the R882 hotspot mutation being the most relevant [66]. Later-scale population-based analyses, including statistical modeling of the UK Biobank data, have further identified *DNMT3A* mutations as a significant risk factor for the development of AML [67,68]. Mechanistically, the *DNMT3A^R882H^* mutation, located within the catalytic domain, exerts a dominant-negative effect by impairing the formation of enzymatically active DNMT3A tetramer. This results in reduced methyltransferase activity and locus-specific DNA hypomethylation, leading to repression of key differentiation-associated transcription factors such as *CEBPA* and *PU.1* [69,70]. In addition, *DNMT3A^R882H^* preferentially induces hypomethylation at polycomb repressive complex 2 (PRC2) target regions, and promotes aberrant activation of self-renewal programs, including enhanced binding at *MYC*-associated motifs [71].

In contrast, TET2 functions as a DNA demethylation enzyme that catalyzes the oxidation of 5-methylcytosine and thereby acts as a key regulator of DNA methylation dynamics. Loss-of-function mutations in *TET2* are detected in approximately 15% of patients with myeloid malignancies [72]. Mechanistically, TET2 shapes enhancer methylation landscapes and cooperates with lineage-defining TFs, such as PU.1 and RUNX1, to regulate myeloid differentiation [73,74]. Recent work using *Tet2* knockout mouse models identified SOX4 as a critical downstream driver, activating NOTCH, FGF and PI3K signaling pathways and conferring selective clonal advantage contributing to *Tet2*-deficient hematopoietic cells [75].

**Figure 3:**
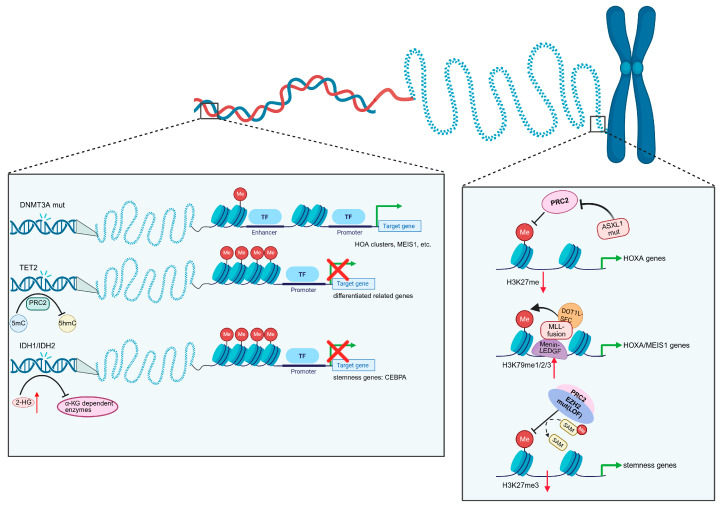
Interplay of epigenetic and transcriptional dysregulation in leukemia. Left panel depicts DNA-methylation-related abnormalities, where mutations in DNMT3A, TET2, or IDH1/2 alter DNA methylation states, affecting enhancer–promoter interactions and transcription factor binding. These changes collectively result in inappropriate activation of leukemogenic programs and repression of differentiation-associated genes. Right panel shows alterations in histone modification pathways, including ASXL1-mutation-disrupted PRC2-mediated H3K27 trimethylation; aberrant activation of MLL fusion complexes leads to increased H3K79 methylation and dysregulated expression of HOXA/MEIS1 and stemness-associated genes; EZH2 is the core component of PRC2; its mutation causes reduced H3K27mes. In the figure, green arrows indicate activation, red crosses denote gene repression or loss of function, red upward arrows indicate upregulation, whereas red downward arrows indicate downregulation. Different colored shapes represent specific proteins, complexes, or epigenetic modifications (e.g., methylation marked as “Me”). Created in BioRender. Yang, J. (2026) https://BioRender.com/hh0kzwt (accessed on 22 December 2025).

Mutations of isocitrate dehydrogenase (IDH) genes were first identified in 2009 through AML genome sequencing [76]. IDH1 and IDH2 encode metabolic enzymes that link cellular metabolism with epigenetic regulation. Recurrent mutations in *IDH1 (R132)* and *IDH2 (R140, R172)* result in neomorphic enzyme activity that produces the oncometabolite 2-hydroxyglutarate (2-HG) instead of α-ketoglutarate (α-KG). Accumulation of 2-HG competitively inhibits α-KG-dependent dioxygenases, including TET family enzymes and lysine histone demethylases, leading to impaired DNA and histone demethylation and global DNA hypermethylation [77,78,79,80]. CHIP-seq analyses have further revealed that IDH^mut^-specific hypermethylation is enriched at active enhancers regions, including loci associated with *MYC* and *ETV6*, which directly interact with key AML oncogenic programs [81]. Moreover, 2-HG inhibits α-KG-dependent alkB homolog (ALKBH) DNA repair enzymes, resulting in increased DNA damage accumulation and contributing to genomic instability [82]. Collectively, IDH mutations disrupt epigenetic homeostasis and DNA repair mechanisms in HSCs, thereby impairing cell differentiation and promoting dysregulated proliferation that drives leukemogenesis (Figure 3, left box).

### 5.2. Histone Modifications and Chromatin Remodeling

Mutations in genes regulating histone modifications disrupt chromatin organization and represent key drivers of AML. Among these, ASXL1, EZH2, and KMT2A are frequently mutated and lead to profound alterations in transcriptional programs controlling hematopoietic differentiation and self-renewal.

*ASXL1* mutations result in a global reduction in H3K27me3 by impairing recruitment of the polycomb repressive complex2 (PRC2) to oncogenic loci, including the *HOXA* cluster, thereby promoting myeloid transformation [83]. In addition, ASXL1 interacts with O-GlcNAc transferase (OGT) to regulate H3K4 methylation, and disruption of this axis impairs normal myeloid differentiation [84]. EZH2, the catalytic subunit of PRC2, mediates H3K27me3-dependent gene repression and exhibits context-dependent roles in cancer. While gain-of-function *EZH2* mutations in lymphomas enhance silencing of tumor suppressor genes [85,86], loss-of-function *EZH2* mutations are common in myeloid malignancies. In AML, EZH2 functions as a tumor suppressor during disease initiation by repressing fetal oncogenic programs such as *PLAG1*, but later supports disease maintenance by sustaining repression of differentiation-associated genes and leukemic stem cell programs [87,88]. *KMT2A*, also known as *MLL1*, encodes a histone methyltransferase that catalyzes H3K4me3 and regulates *HOX* gene transcription through interactions with the Menin–LEDGF complex [89,90,91]. In *KMT2A*-rearranged leukemias, the C-terminal SET domain is replaced by fusion partners such as *AFF1*, *MLLT3*, *MLLT10*, *MLLT1*, or *ELL* [91]. These fusion proteins retain chromatin-targeting activity and aberrantly recruit transcriptional elongation machinery and DOT1L to *HOXA*/*MEIS1* loci, leading to increased H3K79 methylation, sustained oncogene expression, and leukemic self-renewal [89,92,93] (Figure 3, right box).

Dysregulation of PRCs further alter chromatin states in AML. PRC1-mediated H2AK119 ubiquitination and PRC2-mediated H3K27me3 cooperate to repress differentiation-associated genes, thereby maintaining leukemic self-renewal [93,94]. Increased activity of PRC1 components RING1A and RING1B enhances repression of differentiation programs in AML [95,96]. PRC2 consists of EZH1/2, SUZ12, EED, and RBBP4/7, and AML-associated mutations in these subunits destabilize the complex and reduce H3K27me3 levels [94]. This leads to de-repression of stemness-related genes such as *HOXA9*, *MEIS1*, and *PLAG1*, promoting leukemogenesis [87,88,94]. Many PRC target genes exhibit bivalent chromatin marked by both H3K4me3 and H3K27me3; disruption of this balance maintains leukemic cells in a differentiation-blocked, stem-like state [97,98].

Aberrant activation of super-enhancers also plays a central role in AML. Leukemia cells hijack super-enhancer machinery involving transcription factors, p300/CBP, BRD4, and SWI/SNF to drive oncogenes such as *MYC* and promote self-renewal [99,100,101,102,103]. Chromosomal rearrangements can rewire enhancer landscapes, as seen in inv(3)/t(3;3) AML, where a *GATA2* enhancer activates *EVI1* while causing *GATA2* haploinsufficiency [104]. Additionally, TRIB1 remodels *Hoxa9*-associated super-enhancers by promoting C/EBPα degradation, increasing H3K27ac and oncogenic transcription [105]. The super-enhancer-associated gene *CAPG* promotes AML progression through NF-kB signaling and is associated with poor prognosis [101]. Although super-enhancers are clearly critical for AML pathogenesis, their therapeutic targeting and interactions with other genetic lesions remain areas of active investigation.

### 5.3. Noncoding RNAs and RNA Modifications

Noncoding RNAs (ncRNAs) have emerged as critical regulators of gene expression and play integral roles in the pathogenesis of AML. Based on transcript length and structural features, ncRNAs are broadly classified into microRNAs (miRNAs), long noncoding RNAs (lncRNAs) and circular RNAs (circRNAs).

miRNAs are small, ~22-nucleotide RNA molecules that post-transcriptionally repress gene expression by binding to complementary sequences within the 3′ untranslated region (3′-UTRs) of target mRNAs. Early profiling studies revealed distinct, AML subtype-specific miRNA expression signatures that are closely associated with leukemogenesis and clinical outcomes [106,107,108]. In *FLT3-ITD* AML, miR-155 is transcriptionally induced by p65 and STAT5, subsequently suppressing the myeloid transcription factor *PU.1* and thereby impairing myeloid differentiation [109,110]. miR-196b exerts a complex- and stage-dependent function in MLL-rearranged leukemia and normal hematopoiesis by simultaneously targeting oncogenic factors (*HOAX9*/*MEIS1*) and the proapoptotic tumor suppressor *FAS* [111,112]. Notably, miRNAs can exhibit opposing roles depending on the AML subtype. For example, miR-9 functions as an oncogene in *MLL/AF9*-driven leukemia [106], whereas in pediatric AML harboring t(8;21) translocations or *EVI1* overexpression, miR-9 acts as a tumor suppressor by inhibiting leukemic progress and promoting differentiation [113,114].

circRNAs are covalently closed, highly stable RNA molecules generated through back-splicing during pre-mRNA processing. Emerging evidence indicates that dysregulated circRNA expression contributes to AML pathogenesis and may serve as a prognostic biomarker or therapeutic target [115]. Bioinformatic analyses have identified circ-0004277 as one of the most significantly downregulated circRNAs in AML [116]. Fusion gene-driven -circRNAs, including *PML/RARα*-driven f-circPR and *MLL/AF9*-derived f-circM9, promote leukemia cell survival and disease progression [117]. Furthermore, exosomal circ_0006896 has been shown to interact with HDAC1, leading to reduced H3 acetylation, suppression of lipid peroxidation and inhibition of ferroptosis in AML cells [118].

lncRNAs are transcripts longer than 200 nucleotides that regulate gene expression through diverse mechanisms, including chromatin remodeling, transcriptional regulation, and modulation of RNA stability and translation. HOTAIRM1, a well-characterized lncRNA transcribed from the *HOXA* cluster, plays a pivotal role in myeloid differentiation [119,120]. In addition, several lncRNAs, such as UCA1, NEAT, MEG3, and MALAT1, have been shown to function either as oncogenes or tumor suppressors in AML, depending on cellular context and genetic background [121,122].

Collectively, ncRNAs constitute a complex and multilayered regulatory network that orchestrates gene expression programs for AML initiation, maintenance, and progression. Elucidation of ncRNA-mediated regulatory mechanisms not only deepens our understanding of AML biology but also offers foundation for the development of ncRNA-based diagnostic indictors and targeted therapeutic strategies.

Beyond ncRNAs, N6-methyladenosine (m^6^A) RNA modification has emerged as an additional critical epitranscriptomic layer of gene regulation in AML [123,124,125]. m^6^A modification is dynamically regulated by methyltransferase complexes (writers), such as METTL3 and METTL14; demethylases (erasers), including FTO; and m^6^A-binding proteins (readers), such as YTHDC1/2, YTHDF2, and IGF2BP2. METTL3 is frequently overexpressed in AML and sustains leukemic cell proliferation while blocking myeloid differentiation by enhancing m^6^A-dependent translation of key oncogenic transcripts, including MYC, BCL2, and SP1 [126,127]. Conversely, the m^6^A demethylase FTO acts as an oncogenic driver by reducing m^6^A levels on target mRNAs, such as ASB2 and RARA, thereby promoting leukemogenic programs [128]. Notably, Wenlong Li and colleagues developed a selective FTO degrader that suppresses AML progression by increasing m^6^A modifications on ribosome-biogenesis-related mRNAs, promoting their YTHDF2-mediated decay, disrupting protein translation and highlighting FTO as a promising therapeutic target in AML [129].

### 5.4. Chromatin Architecture and 3D Genome Organization

Chromatin 3D architecture alternation driven by AML mutations has become a critical layer of epigenetic regulation in recent years. Chromatin is highly hierarchical, organized into A/B compartments, topologically associating domains (TADs), and chromatin loops, largely sustained by architectural proteins including CTCF, cohesion, lamins and the Mediator complex [130,131,132,133]. In AML, accumulating evidence has shown that chromatin architecture is profoundly changed. Hi-C-based studies have revealed AML-specific chromatin loops and expanded, shrunken and shifted TAD boundaries in AML compared to HSPCs. WGS data also showed enhancer hijacking in genes such as HSF4, MYC, CBL and POU2F3, as well as silencer hijacking affecting AML-related genes including JAK1 and KMT2C, which regulate gene expression [132]. Mutations in chromatin architectural components also play role in remodeling the 3D genome. Cohesion component STAG2, which functions as a leukemogenic potential mutation, shapes chromatin interaction through RAD21 and CTCF binding, resulting in increased HSC self-renewal and disturbed differentiation [134].

Importantly, alterations in 3D genome organization are tightly incorporated with other epigenetic regulations, including DNA methylation, histone modification and noncoding-RNA-mediated regulation. Research has demonstrated that genes in the A compartment have lower methylation at transcription starting site (TSS) but higher methylation in gene bodies than genes in the B compartment. The impact of DNA methylation on the 3D genome structure can be reversed by DNMT3A/3B/1 triple-knockdown (TKD) in the U937 AML cell line [132].

Altogether, structural variations, fusion oncoproteins, and mutations in chromatin regulators in AML collectively reshape chromatin topology, thereby stabilizing leukemic transcriptional states, promoting leukemic transcriptional programs, and contributing to leukemogenesis.

## 6. Disrupted Signaling Pathways in AML

Accumulating evidence indicates that dysregulated signaling pathways are fundamental drivers of AML, governing leukemic cell survival, apoptosis, proliferation, and stemness. These pathways play pivotal roles in leukemogenesis and represent key targets for therapeutic intervention.

### 6.1. Proliferation and Survival Pathways

Receptor tyrosine kinases (RTKs) are central regulators of cellular proliferation, survival, apoptosis, and differentiation. Among them, c-KIT and FMS-like tyrosine kinase 3 (FLT3), members of the class III protein tyrosine kinase (PTK) family, are of particular importance in acute myeloid leukemia (AML), as their aberrant activation is frequently associated with adverse clinical outcomes. c-KIT is commonly overexpressed or mutated in AML, while FLT3 internal tandem duplication (FLT3-ITD) mutations represent one of the most prevalent genetic alterations in the disease. Constitutive activation of c-KIT and FLT3-ITD leads to sustained signaling through key oncogenic pathways, including PI3K/AKT, RAS/RAF/MEK/ERK, and JAK/STAT, thereby promoting leukemic cell proliferation, survival, and resistance to apoptosis [48,135,136,137,138]. Among downstream effectors, STAT5 signaling plays a particularly critical role in maintaining leukemia stem cell (LSC) properties. Genetic or pharmacological suppression of STAT5 in CD34^+^ AML cells significantly impair long-term repopulating capacity, underscoring its essential function in LSC self-renewal and disease persistence [139,140].

In addition to RTK-driven signaling, activating mutations in small GTPases of the RAS family constitutes another major oncogenic axis in AML. The canonical RAS gene family includes *KRAS*, *NRAS*, and *HRAS*, with *KRAS* and *NRAS* being the most frequently mutated in AML [141,142]. Oncogenic *RAS* mutations result in constitutive activation of downstream effector pathways, including MAPK and PI3K-AKT signaling, as well as Ral GTPase-mediated processes, collectively driving aberrant cell proliferation, survival, and metabolic adaptation [143,144].

Importantly, AML progression is not solely dictated by intrinsic genetic alterations but is profoundly influenced by the BM microenvironment, which cooperates with oncogenic signaling to support leukemic cell survival and expansion. Through ligand–receptor interactions such as CXCL12/CXCR4, VEGF/VEGFR, and integrin-mediated adhesion, the BM niche robustly activates core signaling pathways, including RAS-MAPK, PI3K-AKT, NF-kB, and mTOR cascades [15,16,17,18,19,20,21,22,23,24,25,26,27,28,29,30,31,32,33,34,35,36,37,38,39,40,41,42,43,44,45,46,47,48,49,50,51,52,53,54,55,56,57,58,59,60,61,62,63,64,65,66,67,68,69,70,71,72,73,74,75,76,77,78,79,80,81,82,83,84,85,86,87,88,89,90,91,92,93,94,95,96,97,98,99,100,101,102,103,104,105,106,107,108,109,110,111,112,113,114,115,116,117,118,119,120,121,122,123,124,125,126,127,128,129,130,131,132,133,134,135,136,137,138,139,140,141,142,143,144,145,146,147]. Upregulation of E-selection in AML bone marrow further enhances leukemic blast survival by stimulating MAPK/ERK and PI3K/AKT signaling [148,149]. Notably, microenvironment-derived cues synergize with recurrent AML mutations, such as *FLT3-ITD*, to amplify downstream signaling output and promote leukemic cell growth and therapy resistance [150,151]. Collectively, the dynamic interplay between intrinsic genetic drivers and extrinsic BM microenvironmental signals converges on shared proliferation and survival pathways, underscoring the niche as a critical co-driver of AML pathogenesis [152,153].

### 6.2. Self-Renewal and Stemness Pathways

The maintenance and malignant expansion of LSCs are critically dependent on the aberrant activation of developmental signaling pathways, such as Wnt/β-catenin, NOTCH, and Hedgehog (Hh) signaling, which collectively confer stemness and self-renewal capacity. Dysregulation of these pathways enables LSCs to sustain long-term leukemic propagation and evade differentiation cues. In the canonical Wnt/β-catenin pathway, stabilization of β-catenin leads to its cytoplasmic accumulation and subsequent nuclear translocation, where it binds to TCF/LEF family transcription factors. This transcriptional complex activates downstream target genes, including *MYC*, *CCND1* and *ABCB1*, thereby promoting LSCs self-renewal, proliferation, and drug resistance [154,155]. Multiple studies have identified epigenetic silencing of Wnt pathway antagonists, such as secreted frizzled-related proteins (SFRPs) and *DKK1*, through promoter hypermethylation as a mechanism driving constitutive Wnt pathway activation in AML [155,156,157]. Pharmacological or genetic inhibition of β-catenin signaling, including modulation of GSK3β activity, significantly reduces AML cell proliferation and impairs LSC self-renewal capacity [158].

The NOTCH signaling pathway is initiated by the interaction between Delta-like (DLL) or Jagged-like (JAG) ligands and Notch receptors, resulting in the release of the Notch intracellular domain and transcriptional activation of downstream effectors. In contrast to its oncogenic role in some hematologic malignancies, NOTCH signaling appears to be attenuated in AML. Reduced expression of canonical NOTCH target genes, including *HES1* and *DTX1*, has been observed in AML, suggesting a state of suppressed NOTCH pathway activity [159]. Epigenetic repression, characterized by increased H3K27 trimethylation at promoters of NOTCH target genes, has been proposed as a major mechanism underlying this suppression. Functionally, NOTCH inhibition promotes expansion of multipotent progenitors, whereas enforced activation of NOTCH signaling restricts AML growth and LSC maintenance, supporting a tumor-suppressive role for NOTCH in AML [159,160].

Aberrant activation of the Hedgehog (Hh) signaling pathway represents another critical mechanism sustaining LSC survival and self-renewal in AML. Canonical Hh signaling culminates in the activation of GLI family transcription factors, which regulate genes involved in stemness, quiescence, and survival [161,162]. Hh signaling not only directly supports LSC self-renewal but also modulates interactions between LSCs and the BM microenvironment, facilitating maintenance of quiescence and protection from chemotherapy-induced cytotoxicity [163,164].

At the transcriptional level, the LSC stemness phenotype is frequently driven by the aberrant expression of key stemness-associated genes, most notably *HOXA9* and *MEIS1*. These Homeobox transcription factors are highly expressed in *MLL*-rearranged AML and other high-risk AML subtypes, including *NPM1*-mutated AML [165,166]. The synergistic action of *HOXA9* and *MEIS1* is essential for maintaining LSC self-renewal, leukemia propagation, and differentiation blockade [167,168]. Therapeutic targeting of this axis has therefore emerged as a promising strategy. Menin inhibitors, such as Revumenib and Ziftomenib, disrupt the Menin–KMT2A interaction, leading to suppression of both *HOXA9* and *MEIS1* transcription and induction of LSC differentiation and leukemic cell apoptosis [58,98,169].

### 6.3. Apoptosis and Cell Cycle Dysregulation

Dysregulation of apoptosis and cell cycle control is a fundamental mechanism underlying leukemogenesis and therapeutic resistance in AML. Under physiological conditions, the tumor suppressor p53 functions as a central cellular stress sensor that preserves genomic integrity by orchestrating cell cycle arrest, DNA repair, apoptosis, or senescence in response to oncogenic or genotoxic stress [170,171]. In AML, however, disruption of the p53 pathway enables leukemic cells to evade apoptosis and bypass cell cycle checkpoints. *TP53* mutations occur in approximately 5~10% of AML cases and are strongly associated with adverse clinical outcomes. AML cells circumvent p53-mediated tumor-suppressive functions primarily through two mechanisms. First, *TP53* mutations result in the accumulation of transcriptionally inactive p53 protein, which correlates with poor prognosis. Second, functional inactivation of p53 frequently occurs in the absence of *TP53* mutations through overexpression of its negative regulators, MDM2 and MDM4. Notably, MDM2 overexpression can phenocopy TP53 loss and is associated with similarly unfavorable outcomes. Consistent with impaired p53 signaling, a substantial proportion of AML samples exhibit loss of p21 expression, directly reflecting disruption of p53-dependent cell cycle arrest [172,173,174].

Apoptotic signaling in AML is predominantly regulated by the BCL-2 family of proteins, which governs the intrinsic mitochondrial apoptotic pathway. This family comprises anti-apoptotic members (including BCL-2, BCL-XL, MCL-1, BCL-B, BCL2A1, and BCL-W) and pro-apoptotic members, which include the multidomain effectors BAX and BAK, as well as BH3-only proteins such as PUMA, NOXA, BIM, BID, BAD, BIK, and others. The balance between these opposing factions determines cell fate. BH3-only activator proteins (e.g., BID, BIM, PUMA, and NOXA) directly activate BAX and BAK, leading to mitochondrial outer-membrane permeabilization (MOMP), cytochrome c release, caspase activation, and execution of apoptosis. In contrast, BH3-only sensitizer proteins (e.g., BAD, BIK, and HRK) promote apoptosis indirectly by sequestering anti-apoptotic BCL-2 family members, thereby liberating activator proteins to engage BAX and BAK [175,176].

Early studies demonstrated that BCL-2 is frequently overexpressed in AML and is associated with poor prognosis and chemo-resistance [177]. Therefore, pharmacological targeting of BCL2 has emerged as a highly effective therapeutic strategy. Small-molecule BCL2 inhibitors, such as venetoclax, function as BH3 mimetics that displace BAX/BAK from anti-apoptotic sequestration, thereby restoring mitochondrial apoptotic signaling and inducing leukemic cell death [178,179]. Nevertheless, both primary and acquired resistance remain significant clinical challenges, arising from compensatory mechanisms, such as upregulation of alterative anti-apoptotic proteins (MCL1 and BCL-xL), mitochondrial reprogramming, and metabolic adaptions that sustain leukemic cell survival [180,181,182].

## 7. Leukemic Stem Cells and Intratumoral Heterogeneity

In AML, LSCs originate from diverse oncogenic drivers and pre-LSCs populations, resulting in substantial genetic, epigenetic and cellular heterogeneity. This heterogeneity is closely associated with differential responses to targeted therapies, variable disease trajectories, adverse clinical outcomes, and frequent relapse. Consequently, a detailed characterization of the phenotypic and functional properties of LSCs is critical for defining distinct LSC subtypes and for enabling precision-based therapeutic strategies in AML [183,184].

### 7.1. Phenotypic Heterogeneity

Historically, AML LSCs have been operationally defined by a CD34^+^CD38^−^ immunophenotype [185]. However, increasing evidence indicates that LSCs are highly heterogeneous and exhibit marked phenotypic plasticity. A broad spectrum of additional surface markers, including CD123, CD33, TIM3, CD47, CLL-1, CD25, CD44, CD96, and GPR56, has been identified across multiple studies [41,42,43,44,45,46,186,187,188]. Notably, CD123 and CD33 are highly expressed on AML blasts and LSCs, and are associated with inferior remission rates; over 90% of AML patients express at least one of these antigens [189]. Therapeutic targeting of CD33 or CD123 using T-cell engager (TCE) antibodies and chimeric antigen receptor (CAR)–T cell approaches has demonstrated clinical efficacy [190,191,192]. T-cell immunoglobulin mucin-3 (TIM-3) has emerged as a functional marker, identifying a residual LSC population that persists following hematopoietic stem cell transplantation and is strongly predictive of relapse, underscoring its prognostic and functional relevance [193]. In addition, CD47 functions as an innate immune checkpoint that is upregulated in LSCs, enabling evasion of macrophage-mediated phagocytosis and adding further complexity to LSC biology [194,195]. Collectively, this extensive immunophenotypic diversity complicates the unequivocal identification of LSCs while simultaneously providing opportunities for refined diagnostic stratification and targeted therapeutic intervention.

### 7.2. Functional and Metabolic Heterogeneity

Beyond phenotypic features, LSCs are distinguished by their unique functional properties. A hallmark characteristic of LSCs is their propensity to reside in a quiescent or dormant state, which confers resistance to cytotoxic chemotherapy and targeted agents. These quiescent LSCs can subsequently re-enter the cell cycle, contributing to minimal residual disease (MRD) persistence and disease relapse [185,196]. Mechanistically, LSC dormancy is governed by a multilayered regulatory network integrating metabolic control with transcriptional and epigenetic programs. LSCs exhibit pronounced metabolic heterogeneity; quiescent LSCs preferentially rely on oxidative phosphorylation (OXPHOS) rather than glycolysis, thereby minimizing energy expenditure [197]. This metabolic state is accompanied by a distinctive mitochondrial phenotype characterized by reduced mitochondrial mass, altered morphology, and dynamic remodeling, which collectively limit reactive oxygen species (ROS) production and preserve stemness [198]. Furthermore, elevated expression of the anti-apoptotic protein BCL-2 supports mitochondrial respiration in LSCs. Pharmacological inhibition of BCL-2 with venetoclax disrupts OXPHOS and selectively eliminates quiescent LSCs, highlighting mitochondrial metabolism as a critical therapeutic vulnerability in AML [199]. LSCs also exhibit a pro-inflammatory phenotype, express fatty acid transporter CD36, and induce lipolysis in BM adipocytes to fuel fatty acid oxidation (FAO) in leukemic cells [200].

**Figure 4:**
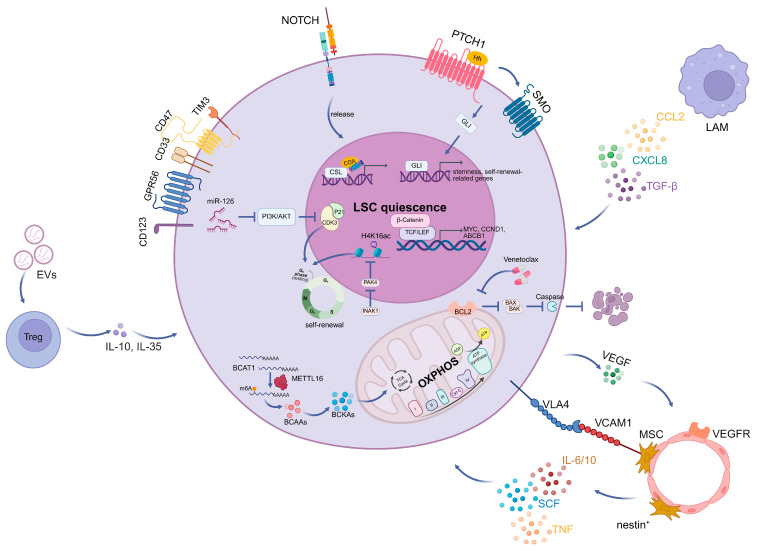
Multilayered regulation of leukemic stem cell (LSC) quiescence. Within the LSC, transcriptional and epigenetic programs converge to sustain a quiescent, stem-like state. NOTCH and GLI signaling activate stemness- and self-renewal-associated gene expression. The Wnt signaling pathway shows stabilization of β-catenin leading to its cytoplasmic accumulation and subsequent nuclear translocation, where it binds to TCF/LEF family transcription factors, thereby activating downstream genes. miRNA-126 restrains the cell cycle through the PI3K-AKT pathway. INKA1 also restricts PAK4 nuclear localization, thereby limiting global H4K16 acetylation and maintaining LSCs in a primitive G0 state. Metabolic adaptations further support LSC quiescence, with a preferential reliance on mitochondrial oxidative phosphorylation (OXPHOS) rather than glycolysis. Anti-apoptotic signaling through BCL-2 preserves mitochondrial integrity and energy homeostasis, promoting LSC survival. METTL16 reprograms BCAA metabolism by driving the m^6^A-dependent expression of branched-chain amino acid (BCAA) transaminases BCAT1 and NCAT. Interactions with bone marrow niche components, such as mesenchymal stromal cells (MSCs), endothelial cells, regulatory T cells (Tregs), and leukemia-associated macrophages (LAMs), provide soluble factors (e.g., SCF, IL-6/10, TNF, VEGF, TGF-β, CXCL8, and CCL2) and adhesion-mediated signals (e.g., VLA4-VCAM1) that reinforce LSC dormancy and survival. Created in BioRender. Yang, J. (2026) https://BioRender.com/hh0kzwt (accessed on 22 December 2025).

Increasing evidence also underscores the pivotal role of gene regulatory networks in enforcing LSC quiescence. INKA1, also known as C3orf54, functions as a key regulator by restraining the G0 phase and delaying early repopulation while preserving long-term self-renewal capacity. Mechanistically, INKA1 impairs the nuclear localization of PAK4, leading to reduced global H4K16 acetylation and maintenance of LSCs in a primitive, quiescent state [201]. Similarly, Forkhead box M1 (FOXM1) has been shown to regulate LSC quiescence through activation of the Wnt-β-catenin signaling pathway in MLL/AF9-driven AML models [202]. MicroRNAs represent an additional layer of post-transcriptional regulation. In particular, miR-126 is highly enriched in LSCs and suppresses AKT activity and CDK3 expression; the miR-126-CDK3 regulatory axis governs G0 exit kinetics and promotes chemotherapy resistance by sustaining LSC quiescence [203]. Moreover, RNA modification enzymes play crucial roles in LSC maintenance. METTL3 and METTL14 promote cell cycle arrest in LSCs [126,204,205], whereas METTL16 drives the m^6^A-dependent expression of branched-chain amino acid (BCAA) transaminases BCAT1 and NCAT2, thereby reprogramming BCAA metabolism in AML [206] (Figure 4).

### 7.3. Microenvironmental Heterogeneity

LSC harbors a highly heterogenous bone marrow environment (BMM), where spatially and functionally distinct niches exert differential control over LSC fate. Single-cell and spatial transcriptomic analyses in recent years have revealed that AML bone marrow is composed of multiple mesenchymal stromal, endothelial, and immune subpopulations with non-redundant roles in supporting leukemic growth and persistence.

Spatially, LSCs occupy distinct niches within BM. Primitive, quiescent LSCs are preferentially enriched in endosteal and arteriolar regions, which are characterized by low oxygen tension and enriched in niche-derived quiescence signals. In contrast, more proliferative LSC subsets are localized predominantly to perivascular and sinusoidal niches, where higher oxygen availability and cytokine concentrations support metabolic activity and clonal expansion. This spatial heterogeneity generates differential exposure to extrinsic cues, resulting in coexisting LSC populations with distinct functional states within the same patient.

At the cellular level, the BM niche supporting AML LSCs is composed of diverse stromal and non-stromal cell types. Mesenchymal stromal cells (MSCs) and endothelial cells provide key factors such as CXCL12 and stem cell factor (SCF) that promote LSC survival and self-renewal [23]. Osteoblast lineage cells have been implicated in maintaining quiescent, therapy-resistant LSC pools [25], whereas adipocytes support LSCs through enhancing fatty acid oxidation (FAO) [201,207]. In addition, immune cells within the niche, including macrophages and regulatory T cells, contribute to immune evasion and protection of LSCs from cytotoxic therapies [208,209].

Importantly, niche heterogeneity is highly dynamic. AML cells actively remodel the BM microenvironment by altering stromal cell function, vascular integrity, and cytokine gradients, thereby establishing leukemia-permissive niches. This dynamic interplay between LSCs and heterogeneous BM niches underscores the critical role of microenvironmental context in sustaining LSC plasticity and driving disease persistence.

## 8. Bone Marrow Microenvironment and Immune Interactions

The bone marrow microenvironment (BMM) constitutes a highly specialized and dynamic niche that orchestrates normal hematopoiesis through tightly regulated interactions among vascular, stromal, and immune components. In AML, leukemic blasts and leukemia stem cells (LSCs) actively remodel this niche to establish a heterogeneous and permissive environment that supports leukemic survival, self-renewal, and therapy resistance.

Vascular remodeling represents a hallmark of AML-induced niche alterations [210]. Leukemic cells drive aberrant angiogenesis by secreting pro-angiogenic factors such as VEGF [211], adhesion molecules including VCAM-1 [212] and E-selection [213,214], and inflammatory cytokines such as TNF-α, collectively resulting in an expanded yet dysfunctional BM vasculature [210,215,216]. This dysregulated vascular network provides protective niches that shield leukemic cells from chemotherapeutic insult via activation of AKT/mTOR and NF-kB pathways [214]. Concurrently, endothelial cells exposed to AML cells undergo phenotypic changes, further reinforcing leukemic cell adhesion and quiescence [216].

Stromal components, particularly mesenchymal stromal cells (MSCs), are profoundly reprogrammed in AML. Leukemia-educated MSCs exhibit altered transcriptional reprogramming, including upregulation of E-cadherin [217], induction of connective tissue growth factor [218], reduced capacity to support normal hematopoiesis, and increased secretion of leukemogenic cytokines and chemokines such as SCF, TNF, IL6, IL-10 and M-CSF [217,219,220,221,222]. Additionally, nestin^+^ BMSCs enhance OXHPOS, TCA activity and GSH-mediated antioxidant defense, thereby promoting AML survival and chemotherapy resistance [223]. Furthermore, nestin^+^ BMSCs facilitate AML relapse by sustaining high protein translation in chemo-resistant leukemia cells through the transfer of cap-dependent translation machinery, including eIF4A, via extracellular vesicles [224]. MSCs also contribute to AML survival and drug resistance via HDAC3-driven mitochondrial ROS accumulation, which induces stromal senescence and generates a hyperinflammatory niche that activates NF-kB signaling in leukemia cells. Collectively, AML-derived signals disrupt osteoblastic differentiation, metabolic capacity and bone homeostasis, thereby eroding HSC-supportive niches and facilitating leukemic dominance [218,225,226,227,228].

Immune remodeling is another critical dimension of AML-induced niche reprogramming. AML progression is associated with impaired immune surveillance and the accumulation of immunosuppressive cell populations, including regulatory T cells (Tregs), myeloid-derived suppressor cells (MDSCs), leukemia-associated macrophages (LAMs), regulatory B cells (Bregs) and leukemia-associated neutrophils [229,230]. Increased Treg frequency in AML promotes leukemic progression and contributes to therapeutic resistance [231]. Extracellular vesicles (EVs) play a critical role in Treg expansion. Rab27a-dependent secretion of leukemic EVs promotes engraftment and selectively induces effector Treg proliferation. Tregs internalize EVs carrying the costimulatory ligand 4-1BBL (CD137L), which enhances FOXP3 expression and drives an effector Treg phenotype by activating STAT5 signaling while concurrently suppressing mTOR-S6 signaling [232]. EVs can also deliver miRNAs such as miR-21 to promote Treg gene expression [233]. The PD-L1/PD-1 axis further mediates Treg expansion; PD-L1 engagement converts naïve T cells into Tregs by inhibiting AKT-mTOR signaling, whereas PD-L1 blockade attenuates Treg generation [234,235,236]. Additionally, elevated indoleamine 2,3-dioxygenase (IDO) expression in AML, regulated via JAK-STAT1 signaling, produces immunoregulatory metabolites such as kynurenines that further drive Treg expansion [237,238,239,240,241]. Tregs exert immunosuppressive effects through both contact-dependent mechanisms mediated by molecules such as CTLA-4 and NRP-1 [242,243], and contact-independent pathways involving cytokines such as IL-10 and IL-35 [208,244,245]. Additionally, Tregs utilize perforin and granzyme B to induce apoptosis in NK cells and CD8^+^ T cells [246].

Leukemia-associated macrophages (LAMs) are central regulators of the AML niche, producing a spectrum of mediators that influence leukemic progression. Macrophages can polarize into anti-leukemic M1 or pro-leukemic M2 phenotypes, with M2 macrophages being enriched in AML and characterized by surface markers including CD206, CD163, and CD115 [209,247,248,249]. Cytokines such as IL-4 and IL-13 drive M2 polarization primarily through the STAT6 pathway [152], and macrophage colony-stimulating factor (M-CSF) further skews macrophages towards M2-like states [249,250,251]. Additionally, pro-M2 factors, including Gfi1 and let-7b, are upregulated in AML and reinforce the M2 phenotype [252,253]. Metabolically, the M2 phenotype preferentially relies on fatty acid oxidation (FAO) and OXPHOS rather than glycolysis, contains increased mitochondrial content, and utilizes glutamine to fuel TCA cycles [254,255,256,257]. Functionally, M2-like macrophages secret soluble factors such as CCL2, CXCL8, and TGF-β, which enhance leukemic cell survival and suppress apoptosis [209,258,259]. Moreover, immune-checkpoint molecules expressed on tumor-associated macrophages, including PD-L1, TIM-3, and CD47, inhibit anti-tumor immunity and promote leukemia cell persistence. Therefore, LAMs emerge as key contributors to immune evasion and represent promising therapeutic targets for enhancing the efficacy of checkpoint-based immunotherapies [195,229,230,260,261] (Figure 4).

## 9. Emerging Concepts and Future Directions

Recent advances in AML research have been propelled by the rapid development and application of integrative multi-omics technologies, encompassing genomics, epigenomics, transcriptomics, proteomics, and metabolomics. These approaches have substantially refined our understanding of leukemogenesis and disease progression. Large-scale multi-omics studies have demonstrated that integrating multiple molecular layers enables superior risk stratification and the identification of novel molecular dependencies that remain undetectable in single-omics analysis [262,263]. For example, comprehensive inflammatory proteomic profiling of AML patient samples identified oncostatin M receptor (OSMR) as a novel prognostic biomarker [264]. Similarly, pharmacogenomics and epigenomics analysis revealed the efficacy of Menin inhibitors in *KMT2A*-rearranged AML [59].

Single-cell technologies have further elucidated extensive intratumoral heterogeneity, the evolutionary dynamics underlying therapeutic resistance and functional diversity within AML. These methods have uncovered rare subpopulations, including leukemia stem cells (LSCs), therapy-persistent cells, and transient cell states that are often obscured in bulk analyses [262,265]. Single-cell multi-omics profiling has delineated three clonal evolution patterns (monoclonal, linear and branched polyclonal) in complex-karyotype AML (CK-AML) [266]. Single-cell metabolic analysis, using innovative methods such as SCENITH, a flow-cytometry-based method to functionally profile energy metabolism with single-cell resolution [267], have revealed interactions between leukemia redox metabolism and EV signature, thereby providing a more comprehensive metabolic landscape in AML [268].

Single-cell spatial transcriptomics have added an additional layer of resolution by enabling the precise mapping of leukemic cells within their native BM microenvironment. Studies demonstrate that primitive-like AML cells localize near the endosteal niche in BM, whereas committed-like and granulocyte–monocyte progenitor (GMP)-like cells reside more distally. Niche-specific signaling pathways, including CXCL12-CXCR4 and PI3K/AKT/mTOR, regulate leukemic cell differentiation, migration and extramedullary infiltration [269]. Moreover, integrating single-cell spatial transcriptomics with ligand–receptor interaction analysis has revealed that immunotherapy reshapes the spatial organization of leukemic cells and the local enrichment of specific immune cell populations [270].

In addition, genome-wide CRISPR-Cas9 screening has provided causal insights into AML biology by systematically identifying genes and pathways essential for leukemic cell survival and differentiation blockade. For instance, Fermt3, a master regulator of integrin signaling identified in *MLL*-rearranged AML, exhibits significant prognostic relevance [271], while the RNA-binding protein ZFP36L2 has been recognized as a critical regulator of AML maintenance [272]. CRISPR/Cas9 screening has also facilitated the identification of gene markers predictive of chemo-resistance or sensitivity to ADE (cytarabine, daunorubicin, etoposide) chemotherapy in pediatric AML, offering potential biomarkers and therapeutic targets to overcome drug resistance [273].

Collectively, the integration of muti-omics has paved the way for precision immunotherapy and advanced computational modeling. Emerging concepts such as digital twins and generative AI enable patient-specific therapeutic optimization [274,275]. Deep learning frameworks, including the Transcription Regulator Activity Prediction Tool (TRAPT), provide innovative strategies for identifying transcriptional regulators (TRs) [276]. AML digital twins represent a computational paradigm that models individual disease trajectories and therapeutic responses by integrating multi-omics data with clinical and longitudinal treatment information. Unlike traditional static models, digital twins are continuously updated with new data, supporting adaptive treatment optimization and true precision medicine [277,278].

However, several limitations currently hinder their clinical translation. For the multi-omics datasets, precise delineation of cell boundaries, difference in platforms, batch effects and sample bias limit reproducibility and interpretability. From a practical perspective, many advanced multi-omics technologies remain costly and technically demanding for sample quality and processing, which constrains their feasibility for routine clinical. In addition, a substantial proportion of current discoveries are derived from retrospective analyses or relatively small patient cohorts, necessitating validation in large, multicenter, and prospective clinical studies before their utilization as diagnostic, prognostic, or predictive biomarkers can be firmly established. Emerging computational paradigms, including digital twins and generative artificial intelligence, hold great promise for patient-specific therapeutic optimization. Moreover, these approaches are highly dependent on data quality, longitudinal sampling, and model generalizability. Issues related to algorithm interpretability, regulatory approval, and clinical trust further limit their immediate adoption. At present, most AML digital twin frameworks remain at the proof-of-concept stage and require extensive clinical validation.

## 10. Conclusions

Acute myeloid leukemia (AML) is a blood cancer driven by genetic and epigenetic changes that disrupt normal hematopoiesis, allowing leukemic stem cells to thrive. These cells are organized hierarchically and rely on signals from the bone marrow microenvironment to maintain self-renewal, resist therapy, and drive disease progression. Contemporary AML research increasingly leverages integrative systems biology approaches, including multi-omics profiling, single-cell and spatial analyses, and computational modeling, to dissect the mechanisms by which molecular programs, cellular states, and clonal dynamics are established and maintained over time, moving beyond purely descriptive characterization. These insights facilitate the rational design of therapies that address cellular heterogeneity and microenvironmental dependencies, offering broader implications for understanding fundamental principles of cancer biology.

## Figures and Tables

**Table 1:** Functional classes of recurrent AML driver alterations with pathway-specific roles.

Functional Class (NEJM 2013) [29]	Key Genes/Recurrent Alterations
Transcription factor fusions	*PML-RARA*
	*RUNX1-RUNX1T1 (t(8;21))*
	*CBFB-MYH11 (inv(16))*
	*PICALM-MLLT10*
*NPM1 mutations*	*NPM1* (exon 12 frameshift insertions; *NPM1c*)
Tumor suppressor genes	*TP53*
	*WT1*
	*PHF6*
DNA methylation regulators	*DNMT3A* (often *R882* hotspot)
	*DNMT3B*
	*DNMT1*
	*TET1*/*TET2*
	*IDH1 (R132)*
	*IDH2 (R140* vs. *R172)*
Activated signaling pathways	*FLT3*
	*KIT*
	Ser/Thr kinases: *MAPK1*, *BUB1*, *SMG1*, *STK33*, etc.
	*KRAS*/*NRAS*
	PTPs: *PTPN11*, *PTPRT*, *PTPN14*
Myeloid transcription factors	*RUNX1*
	*CEBPA*
Chromatin modifiers	*KMT2A (MLL)* fusions
	*MLL-PTD (KMT2A-PTD)*
	*NUP98-NSD1*
	*ASXL1*
	*EZH2*/*KDM6A*
Cohesion complex genes	*STAG2*, *SMC1A*, *SMC3*, *RAD21*
Spliceosome genes	*SRSF2*, *SF3B1*, *U2AF1*, *ZRSR2*

## Data Availability

No new data were created or analyzed in this study. Data sharing is not applicable to this article.

## References

[1] Ng S.W.K., Mitchell A., Kennedy J.A., Chen W.C., McLeod J., Ibrahimova N., Arruda A., Popescu A., Gupta V., Schimmer A.D. (2016). A 17-gene stemness score for rapid determination of risk in acute leukaemia. Nature.

[2] El Chaer F., Bewersdorf J.P., Stahl M., Zeidan A.M., El Chaer F., Bewersdorf J.P., Stahl M., Zeidan A.M. (2025). The global epidemiology of acute myeloid leukaemia. Nat. Rev. Clin. Oncol..

[3] Gangat N., Dinardo C.D., Gangat N., Dinardo C.D. (2025). Newly diagnosed acute myeloid leukemia in unfit patients: 2026 treatment algorithms. Blood Cancer J..

[4] Eppert K., Takenaka K., Lechman E.R., Waldron L., Nilsson B., van Galen P., Metzeler K.H., Poeppl A., Ling V., Beyene J. (2011). Stem cell gene expression programs influence clinical outcome in human leukemia. Nat. Med..

[5] Khwaja A., Bjorkholm M., Gale R.E., Levine R.L., Jordan C.T., Ehninger G., Bloomfield C.D., Estey E., Burnett A., Cornelissen J.J. (2016). Acute myeloid leukaemia. Nat. Rev. Dis. Primers.

[6] Thomas D., Majeti R. (2017). Biology and relevance of human acute myeloid leukemia stem cells. Blood.

[7] Ediriwickrema A., Gentles A.J., Majeti R. (2023). Single-cell genomics in AML: Extending the frontiers of AML research. Blood.

[8] Tettamanti S., Pievani A., Biondi A., Dotti G., Serafini M. (2022). Catch me if you can: How AML and its niche escape immunotherapy. Leukemia.

[9] Liggett L.A., Sankaran V.G. (2020). Unraveling Hematopoiesis through the Lens of Genomics. Cell.

[10] Buenrostro J.D., Corces M.R., Lareau C.A., Wu B., Schep A.N., Aryee M.J., Majeti R., Chang H.Y., Greenleaf W.J. (2018). Integrated Single-Cell Analysis Maps the Continuous Regulatory Landscape of Human Hematopoietic Differentiation. Cell.

[11] Li H., Côté P., Kuoch M., Ezike J., Frenis K., Afanassiev A., Greenstreet L., Tanaka-Yano M., Tarantino G., Zhang S. (2024). The dynamics of hematopoiesis over the human lifespan. Nat. Methods.

[12] Ye F., Huang W., Guo G., Ye F., Huang W., Guo G. (2017). Studying hematopoiesis using single-cell technologies. J. Hematol. Oncol..

[13] Subramanian S., Thoms J.A.I., Huang Y., Cornejo-Páramo P., Koch F.C., Jacquelin S., Shen S., Song E., Joshi S., Brownlee C. (2023). Genome-wide transcription factor-binding maps reveal cell-specific changes in the regulatory architecture of human HSPCs. Blood.

[14] Wilson N.K., Foster S.D., Wang X., Knezevic K., Schütte J., Kaimakis P., Chilarska P.M., Kinston S., Ouwehand W.H., Dzierzak E. (2010). Combinatorial Transcriptional Control In Blood Stem/Progenitor Cells: Genome-wide Analysis of Ten Major Transcriptional Regulators. Cell Stem Cell.

[15] Frömel R., Rühle J., Martinez A.B., Szu-Tu C., Pastor F.P., Martinez-Corral R., Velten L. (2025). Design principles of cell-state-specific enhancers in hematopoiesis. Cell.

[16] Chou S.T., Khandros E., Bailey L.C., Nichols K.E., Vakoc C.R., Yao Y., Huang Z., Crispino J.D., Hardison R.C., Blobel G.A. (2009). Graded repression of PU.1/Sfpi1 gene transcription by GATA factors regulates hematopoietic cell fate. Blood.

[17] Arinobu Y., Mizuno S.-I., Chong Y., Shigematsu H., Iino T., Iwasaki H., Graf T., Mayfield R., Chan S., Kastner P. (2007). Reciprocal activation of GATA-1 and PU.1 marks initial specification of hematopoietic stem cells into myeloerythroid and myelolymphoid lineages. Cell Stem Cell.

[18] Waldvogel S.M., Camacho V., Fan D., Guzman A.G., Garcia-Martell A., Khabusheva E., Pridgen J.R., De La Fuente J., Rau R., Laidman A.G. (2025). DNMT3A regulates murine megakaryocyte-biased hematopoietic stem cell fate decisions. Blood Adv..

[19] Bröske A.-M., Vockentanz L., Kharazi S., Huska M.R., Mancini E., Scheller M., Kuhl C., Enns A., Prinz M., Jaenisch R. (2009). DNA methylation protects hematopoietic stem cell multipotency from myeloerythroid restriction. Nat. Genet..

[20] López-Moyado I.F., Rao A. (2020). DNMT3A and TET2 mutations reshape hematopoiesis in opposing ways. Nat. Genet..

[21] Moran-Crusio K., Reavie L., Shih A., Abdel-Wahab O., Ndiaye-Lobry D., Lobry C., Figueroa M.E., Vasanthakumar A., Patel J., Zhao X. (2011). Tet2 Loss Leads to Increased Hematopoietic Stem Cell Self-Renewal and Myeloid Transformation. Cancer Cell.

[22] Boulais P.E., Frenette P.S. (2015). Making sense of hematopoietic stem cell niches. Blood.

[23] Greenbaum A., Hsu Y.-M.S., Day R.B., Schuettpelz L.G., Christopher M.J., Borgerding J.N., Nagasawa T., Link D.C. (2013). CXCL12 in early mesenchymal progenitors is required for haematopoietic stem-cell maintenance. Nature.

[24] Ding L., Morrison S.J. (2013). Haematopoietic stem cells and early lymphoid progenitors occupy distinct bone marrow niches. Nature.

[25] Kim M.J., Valderrábano R.J., Wu J.Y. (2022). Osteoblast Lineage Support of Hematopoiesis in Health and Disease. J. Bone Miner. Res..

[26] Ho Y.-H., Toro R.d., Rivera-Torres J., Rak J., Korn C., García-García A., Macías D., González-Gómez C., Monte A.d., Wittner M. (2019). Remodeling of Bone Marrow Hematopoietic Stem Cell Niches Promotes Myeloid Cell Expansion during Premature or Physiological Aging. Cell Stem Cell.

[27] Bandyopadhyay S., Duffy M.P., Ahn K.J., Sussman J.H., Pang M., Smith D., Duncan G., Zhang I., Huang J., Lin Y. (2024). Mapping the cellular biogeography of human bone marrow niches using single-cell transcriptomics and proteomic imaging. Cell.

[28] Weeks L.D., Ebert B.L. (2023). Causes and consequences of clonal hematopoiesis. Blood.

[29] The Cancer Genome Atlas Research Network (2013). Genomic and epigenomic landscapes of adult de novo acute myeloid leukemia. N. Engl. J. Med..

[30] Walter M.J., Shen D., Ding L., Shao J., Koboldt D.C., Chen K., Larson D.E., McLellan M.D., Dooling D., Abbott R. (2012). Clonal Architecture of Secondary Acute Myeloid Leukemia. N. Engl. J. Med..

[31] Mitchell E., Spencer Chapman M., Williams N., Dawson K.J., Mende N., Calderbank E.F., Jung H., Mitchell T., Coorens T.H.H., Spencer D.H. (2022). Clonal dynamics of haematopoiesis across the human lifespan. Nature.

[32] Ryu G., Koh Y., Jaiswal S., Yoon S.-S. (2025). Clonal hematopoiesis: Elements associated with clonal expansion and diseases. Blood Res..

[33] Dunn W.G., McLoughlin M.A., Vassiliou G.S. (2024). Clonal hematopoiesis and hematological malignancy. J. Clin. Investig..

[34] Shlush L.I., Zandi S., Mitchell A., Chen W.C., Brandwein J.M., Gupta V., Kennedy J.A., Schimmer A.D., Schuh A.C., Yee K.W. (2014). Identification of pre-leukaemic haematopoietic stem cells in acute leukaemia. Nature.

[35] Jin L., Wu L. (2023). Recent Advances in Characterization of Pre-Leukemic/Leukemic Stem Cells in Acute Myeloid Leukemia. Cell. Mol. Med. Res..

[36] Wachter F., Pikman Y. (2024). Pathophysiology of Acute Myeloid Leukemia. Acta Haematol..

[37] Petrone G., Turker I., Natarajan P., Bolton K.L. (2024). Clinical and Therapeutic Implications of Clonal Hematopoiesis. Annu. Rev. Genom. Hum. Genet..

[38] Sturgeon C.M., Wagenblast E., Izzo F., Papapetrou E.P. (2024). The Crossroads of Clonal Evolution, Differentiation Hierarchy, and Ontogeny in Leukemia Development. Blood Cancer Discov..

[39] Shin D.-Y. (2022). Human acute myeloid leukemia stem cells: Evolution of concept. Blood Res..

[40] Bonnet D., Dick J.E. (1997). Human acute myeloid leukemia is organized as a hierarchy that originates from a primitive hematopoietic cell. Nat. Med..

[41] van Rhenen A., van Dongen G.A.M.S., Kelder A., Rombouts E.J., Feller N., Moshaver B., Walsum M.S.-V., Zweegman S., Ossenkoppele G.J., Schuurhuis G.J. (2007). The novel AML stem cell associated antigen CLL-1 aids in discrimination between normal and leukemic stem cells. Blood.

[42] Jin L., Lee E.M., Ramshaw H.S., Busfield S.J., Peoppl A.G., Wilkinson L., Guthridge M.A., Thomas D., Barry E.F., Boyd A. (2009). Monoclonal antibody-mediated targeting of CD123, IL-3 receptor alpha chain, eliminates human acute myeloid leukemic stem cells. Cell Stem Cell.

[43] Hosen N., Park C.Y., Tatsumi N., Oji Y., Sugiyama H., Gramatzki M., Krensky A.M., Weissman I.L. (2007). CD96 is a leukemic stem cell-specific marker in human acute myeloid leukemia. Proc. Natl. Acad. Sci. USA.

[44] Majeti R., Chao M.P., Alizadeh A.A., Pang W.W., Jaiswal S., Gibbs K.D., Van Rooijen N., Weissman I.L. (2009). CD47 is an adverse prognostic factor and therapeutic antibody target on human acute myeloid leukemia stem cells. Cell.

[45] Ding Y., Gao H., Zhang Q. (2017). The biomarkers of leukemia stem cells in acute myeloid leukemia. Stem Cell Investig..

[46] Kikushige Y., Shima T., Takayanagi S.-I., Urata S., Miyamoto T., Iwasaki H., Takenaka K., Teshima T., Tanaka T., Inagaki Y. (2010). TIM-3 is a promising target to selectively kill acute myeloid leukemia stem cells. Cell Stem Cell.

[47] Du W., Li X.-E., Sipple J., Pang Q. (2011). Overexpression of IL-3Rα on CD34^+^CD38^−^ stem cells defines leukemia-initiating cells in Fanconi anemia AML. Blood.

[48] Gilliland D.G., Griffin J.D. (2002). The roles of FLT3 in hematopoiesis and leukemia. Blood.

[49] Naoe T., Kiyoi H., Naoe T., Kiyoi H. (2013). Gene mutations of acute myeloid leukemia in the genome era. Int. J. Hematol..

[50] Hoff F.W., Huang Y., Welkie R.L., Swords R.T., Traer E., Stein E.M., Lin T.L., Patel P.A., Collins R.H., Baer M.R. (2025). Molecular characterization of newly diagnosed acute myeloid leukemia patients aged 60 years or older: A report from the Beat AML clinical trial. Blood Cancer J..

[51] Wakita S., Marumo A., Morita K., Kako S., Toya T., Najima Y., Doki N., Kanda J., Kuroda J., Mori S. (2023). Mutational analysis of DNMT3A improves the prognostic stratification of patients with acute myeloid leukemia. Cancer Sci..

[52] Papaemmanuil E., Gerstung M., Bullinger L., Gaidzik V.I., Paschka P., Roberts N.D., Potter N.E., Heuser M., Thol F., Bolli N. (2016). Genomic Classification and Prognosis in Acute Myeloid Leukemia. N. Engl. J. Med..

[53] Chen M., Zeng Z., Li X., Qin W., Cai X., Chen S., Lu X. (2023). Clinical features and prognostic significance of DNMT3A, FLT3, and NPM1 mutations in de novo acute myeloid leukemia patients. Int. J. Lab. Hematol..

[54] Liu P., Liu J.-P., Sun S.-J., Gao Y., Ai Y., Chen X., Sun Y., Zhou M., Liu Y., Xiong Y. (2021). CBFB-MYH11 Fusion Sequesters RUNX1 in Cytoplasm to Prevent DNMT3A Recruitment to Target Genes in AML. Front. Cell Dev. Biol..

[55] Day R.B., Hickman J.A., Xu Z., Katerndahl C.D., Ferraro F., Ramakrishnan S.M., Erdmann-Gilmore P., Sprung R.W., Mi Y., Townsend R.R. (2023). Proteogenomic analysis reveals cytoplasmic sequestration of RUNX1 by the acute myeloid leukemia-initiating CBFB::MYH11 oncofusion protein. J. Clin. Investig..

[56] Testa U., Pelosi E. (2024). Function of PML-RARA in Acute Promyelocytic Leukemia. Transcription Factors in Blood Cell Development.

[57] Swart L.E., Heidenreich O. (2021). The RUNX1/RUNX1T1 network: Translating insights into therapeutic options. Exp. Hematol..

[58] Issa G.C., Aldoss I., DiPersio J., Cuglievan B., Stone R., Arellano M., Thirman M.J., Patel M.R., Dickens D.S., Shenoy S. (2023). The menin inhibitor revumenib in KMT2A-rearranged or NPM1-mutant leukaemia. Nature.

[59] Zehtabcheh S., Soleimani Samarkhazan H., Asadi M., Zabihi M., Parkhideh S., Mohammadi M.H., Zehtabcheh S., Soleimani Samarkhazan H., Asadi M., Zabihi M. (2025). Insights into KMT2A rearrangements in acute myeloid leukemia: From molecular characteristics to targeted therapies. Biomark. Res..

[60] Poitras J.L., Heiser D., Li L., Nguyen B., Nagai K., Duffield A.S., Gamper C., Small D. (2016). Dnmt3a deletion cooperates with the Flt3/ITD mutation to drive leukemogenesis in a murine model. Oncotarget.

[61] Meyer S.E., Qin T., Muench D.E., Masuda K., Venkatasubramanian M., Orr E., Suarez L., Gore S.D., Delwel R., Paietta E. (2016). DNMT3A Haploinsufficiency Transforms FLT3ITD Myeloproliferative Disease into a Rapid, Spontaneous, and Fully Penetrant Acute Myeloid Leukemia. Cancer Discov..

[62] Yang L., Rodriguez B., Mayle A., Park H.J., Lin X., Luo M., Jeong M., Curry C.V., Kim S.-B., Ruau D. (2016). DNMT3A loss drives enhancer hypomethylation in FLT3-ITD-associated leukemias. Cancer Cell.

[63] Ferreira H.J., Heyn H., Vizoso M., Moutinho C., Vidal E., Gomez A., Martínez-Cardús A., Simó-Riudalbas L., Moran S., Jost E. (2015). DNMT3A mutations mediate the epigenetic reactivation of the leukemogenic factor MEIS1 in acute myeloid leukemia. Oncogene.

[64] Ni S.-C., Yao C.-Y., Tsai X.C.-H., Lo M.-Y., Chen C.-Y., Lee W.-H., Lin C.-C., Kuo Y.-Y., Peng Y.-L., Tseng M.-H. (2025). Genomic and transcriptomic determinants of clinical outcomes in patients with AML and DNMT3A mutations. Blood Cancer J..

[65] Huang G., Cai X., Li D. (2024). Significance of targeting DNMT3A mutations in AML. Ann. Hematol..

[66] Ley T.J., Ding L., Walter M.J., McLellan M.D., Lamprecht T., Larson D.E., Kandoth C., Payton J.E., Baty J., Welch J. (2010). DNMT3A mutations in acute myeloid leukemia. N. Engl. J. Med..

[67] Gu M., Kovilakam S.C., Dunn W.G., Marando L., Barcena C., Mohorianu I., Smith A., Kar S.P., Fabre M.A., Gerstung M. (2023). Multiparameter prediction of myeloid neoplasia risk. Nat. Genet..

[68] Weeks L.D., Niroula A., Neuberg D., Wong W., Lindsley R.C., Luskin M., Berliner N., Stone R.M., DeAngelo D.J., Soiffer R. (2023). Prediction of risk for myeloid malignancy in clonal hematopoiesis. NEJM Evid..

[69] Russler-Germain D.A., Spencer D.H., Young M.A., Lamprecht T.L., Miller C.A., Fulton R., Meyer M.R., Erdmann-Gilmore P., Townsend R.R., Wilson R.K. (2014). The R882H DNMT3A mutation associated with AML dominantly inhibits wild-type DNMT3A by blocking its ability to form active tetramers. Cancer Cell.

[70] Koya J., Kataoka K., Sato T., Bando M., Kato Y., Tsuruta-Kishino T., Kobayashi H., Narukawa K., Miyoshi H., Shirahige K. (2016). DNMT3A R882 mutants interact with polycomb proteins to block haematopoietic stem and leukaemic cell differentiation. Nat. Commun..

[71] Nam A.S., Dusaj N., Izzo F., Murali R., Myers R.M., Mouhieddine T.H., Sotelo J., Benbarche S., Waarts M., Gaiti F. (2022). Single-cell multi-omics of human clonal hematopoiesis reveals that DNMT3A R882 mutations perturb early progenitor states through selective hypomethylation. Nat. Genet..

[72] Delhommeau F., Dupont S., Valle V.D., James C., Trannoy S., Massé A., Kosmider O., Le Couedic J.-P., Robert F., Alberdi A. (2009). Mutation in TET2 in Myeloid Cancers. N. Engl. J. Med..

[73] Li Z., Cai X., Cai C.-L., Wang J., Zhang W., Petersen B.E., Yang F.-C., Xu M. (2011). Deletion of Tet2 in mice leads to dysregulated hematopoietic stem cells and subsequent development of myeloid malignancies. Blood.

[74] Joshi K., Zhang L., Breslin S.J.P., Kini A.R., Zhang J. (2022). Role of TET dioxygenases in the regulation of both normal and pathological hematopoiesis. J. Exp. Clin. Cancer Res. CR.

[75] Schiroli G., Kartha V., Duarte F.M., Kristiansen T.A., Mayerhofer C., Shrestha R., Earl A., Hu Y., Tay T., Rhee C. (2024). Cell of origin epigenetic priming determines susceptibility to Tet2 mutation. Nat. Commun..

[76] Mardis E.R., Ding L., Dooling D.J., Larson D.E., McLellan M.D., Chen K., Koboldt D.C., Fulton R.S., Delehaunty K.D., McGrath S.D. (2009). Recurring mutations found by sequencing an acute myeloid leukemia genome. N. Engl. J. Med..

[77] Losman J.-A., Looper R.E., Koivunen P., Lee S., Schneider R.K., McMahon C., Cowley G.S., Root D.E., Ebert B.L., Kaelin W.G. (2013). (R)-2-hydroxyglutarate is sufficient to promote leukemogenesis and its effects are reversible. Science.

[78] Xu W., Yang H., Liu Y., Yang Y., Wang P., Kim S.-H., Ito S., Yang C., Wang P., Xiao M.-T. (2011). Oncometabolite 2-hydroxyglutarate is a competitive inhibitor of α-ketoglutarate-dependent dioxygenases. Cancer Cell.

[79] Glass J.L., Hassane D., Wouters B.J., Kunimoto H., Avellino R., Garrett-Bakelman F.E., Guryanova O.A., Bowman R., Redlich S., Intlekofer A.M. (2017). Epigenetic Identity in AML Depends on Disruption of Nonpromoter Regulatory Elements and Is Affected by Antagonistic Effects of Mutations in Epigenetic Modifiers. Cancer Discov..

[80] Fruchtman H., Avigan Z.M., Waksal J.A., Brennan N., Mascarenhas J.O. (2024). Management of isocitrate dehydrogenase 1/2 mutated acute myeloid leukemia. Leukemia.

[81] Wilson E.R., Helton N.M., Heath S.E., Fulton R.S., Payton J.E., Welch J.S., Walter M.J., Westervelt P., DiPersio J.F., Link D.C. (2022). Focal disruption of DNA methylation dynamics at enhancers in IDH-mutant AML cells. Leukemia.

[82] Wang P., Wu J., Ma S., Zhang L., Yao J., Hoadley K.A., Wilkerson M.D., Perou C.M., Guan K.-L., Ye D. (2015). Oncometabolite D-2-Hydroxyglutarate Inhibits ALKBH DNA Repair Enzymes and Sensitizes IDH Mutant Cells to Alkylating Agents. Cell Rep..

[83] Abdel-Wahab O., Adli M., LaFave L.M., Gao J., Hricik T., Shih A.H., Pandey S., Patel J., Chung Y.R., Koche R. (2012). ASXL1 Mutations Promote Myeloid Transformation Through Loss of PRC2-Mediated Gene Repression. Cancer Cell.

[84] Inoue D., Fujino T., Sheridan P., Zhang Y.-z., Nagase R., Horikawa S., Li Z., Matsui H., Kanai A., Saika M. (2018). A novel ASXL1–OGT axis plays roles in H3K4 methylation and tumor suppression in myeloid malignancies. Leukemia.

[85] Yu H., Hong J., Shin D.-Y., Lee C.-H., Yu H., Hong J., Shin D.-Y., Lee C.-H. (2025). The role of ASXL1, SRSF2, and EZH2 mutations in chromatin dysregulation of myelodysplastic neoplasia and acute myeloid leukemia. Leukemia.

[86] Morin R.D., Johnson N.A., Severson T.M., Mungall A.J., An J., Goya R., Paul J.E., Boyle M., Woolcock B.W., Kuchenbauer F. (2010). Somatic mutations altering EZH2 (Tyr641) in follicular and diffuse large B-cell lymphomas of germinal-center origin. Nat. Genet..

[87] Basheer F., Giotopoulos G., Meduri E., Yun H., Mazan M., Sasca D., Gallipoli P., Marando L., Gozdecka M., Asby R. (2019). Contrasting requirements during disease evolution identify EZH2 as a therapeutic target in AML. J. Exp. Med..

[88] Tanaka S., Miyagi S., Sashida G., Chiba T., Yuan J., Mochizuki-Kashio M., Suzuki Y., Sugano S., Nakaseko C., Yokote K. (2012). Ezh2 augments leukemogenicity by reinforcing differentiation blockage in acute myeloid leukemia. Blood.

[89] Artinger E.L., Mishra B.P., Zaffuto K.M., Li B.E., Chung E.K.Y., Moore A.W., Chen Y., Cheng C., Ernst P. (2013). An MLL-dependent network sustains hematopoiesis. Proc. Natl. Acad. Sci. USA.

[90] Krivtsov A.V., Armstrong S.A., Krivtsov A.V., Armstrong S.A. (2007). MLL translocations, histone modifications and leukaemia stem-cell development. Nat. Rev. Cancer.

[91] Li X., Song Y. (2021). Structure, function and inhibition of critical protein-protein interactions involving mixed lineage leukemia 1 and its fusion oncoproteins. J. Hematol. Oncol..

[92] Wang X., Chen C.-W., Armstrong S.A. (2016). The role of DOT1L in the maintenance of leukemia gene expression. Curr. Opin. Genet. Dev..

[93] Schurer A., Glushakow-Smith S.G., Gritsman K. (2025). Targeting chromatin modifying complexes in acute myeloid leukemia. Stem Cells Transl. Med..

[94] Bhattacharyya T., Bond J. (2023). Polycomb Alterations in Acute Myeloid Leukaemia: From Structure to Function. Cancers.

[95] Nakajima-Takagi Y., Oshima M., Takano J., Koide S., Itokawa N., Uemura S., Yamashita M., Andoh S., Aoyama K., Isshiki Y. (2023). Polycomb repressive complex 1.1 coordinates homeostatic and emergency myelopoiesis. eLife.

[96] Shima H., Takamatsu-Ichihara E., Shino M., Yamagata K., Katsumoto T., Aikawa Y., Fujita S., Koseki H., Kitabayashi I. (2018). Ring1A and Ring1B inhibit expression of Glis2 to maintain murine MOZ-TIF2 AML stem cells. Blood.

[97] Ren Z., Kim A., Huang Y.-T., Pi W.-C., Gong W., Yu X., Qi J., Jin J., Cai L., Roeder R.G. (2022). A PRC2-Kdm5b axis sustains tumorigenicity of acute myeloid leukemia. Proc. Natl. Acad. Sci. USA.

[98] Zhou X., Zhang L., Aryal S., Veasey V., Tajik A., Restelli C., Moreira S., Zhang P., Zhang Y., Hope K.J. (2024). Epigenetic regulation of noncanonical menin targets modulates menin inhibitor response in acute myeloid leukemia. Blood.

[99] Shi J., Whyte W.A., Zepeda-Mendoza C.J., Milazzo J.P., Shen C., Roe J.-S., Minder J.L., Mercan F., Wang E., Eckersley-Maslin M.A. (2013). Role of SWI/SNF in acute leukemia maintenance and enhancer-mediated Myc regulation. Genes Dev..

[100] Roe J.-S., Mercan F., Rivera K., Pappin D.J., Vakoc C.R. (2015). BET Bromodomain Inhibition Suppresses the Function of Hematopoietic Transcription Factors in Acute Myeloid Leukemia. Mol. Cell.

[101] Ma Q., Zhao M., Long B., Li H., Ma Q., Zhao M., Long B., Li H. (2023). Super-enhancer-associated gene CAPG promotes AML progression. Commun. Biol..

[102] Cao Z., Shu Y., Wang J., Wang C., Feng T., Yang L., Shao J., Zou L. (2022). Super enhancers: Pathogenic roles and potential therapeutic targets for acute myeloid leukemia (AML). Genes Dis..

[103] Belloucif Y., Lobry C. (2022). Super-Enhancers Dysregulations in Hematological Malignancies. Cells.

[104] Gröschel S., Sanders M.A., Hoogenboezem R., de Wit E., Bouwman B.A., Erpelinck C., van der Velden V.H., Havermans M., Avellino R., van Lom K. (2014). A single oncogenic enhancer rearrangement causes concomitant EVI1 and GATA2 deregulation in leukemia. Cell.

[105] Yoshino S., Yokoyama T., Sunami Y., Takahara T., Nakamura A., Yamazaki Y., Tsutsumi S., Aburatani H., Nakamura T. (2021). Trib1 promotes acute myeloid leukemia progression by modulating the transcriptional programs of Hoxa9. Blood.

[106] Chen P., Price C., Li Z., Li Y., Cao D., Wiley A., He C., Gurbuxani S., Kunjamma R.B., Huang H. (2013). miR-9 is an essential oncogenic microRNA specifically overexpressed in mixed lineage leukemia-rearranged leukemia. Proc. Natl. Acad. Sci. USA.

[107] Wallace J.A., O’Connell R.M. (2017). MicroRNAs and acute myeloid leukemia: Therapeutic implications and emerging concepts. Blood.

[108] Liu Y., Cheng Z., Pang Y., Cui L., Qian T., Quan L., Zhao H., Shi J., Ke X., Fu L. (2019). Role of microRNAs, circRNAs and long noncoding RNAs in acute myeloid leukemia. J. Hematol. Oncol..

[109] Wallace J.A., Kagele D.A., Eiring A.M., Kim C.N., Hu R., Runtsch M.C., Alexander M., Huffaker T.B., Lee S.-H., Patel A.B. (2017). miR-155 promotes FLT3-ITD-induced myeloproliferative disease through inhibition of the interferon response. Blood.

[110] Gerloff D., Grundler R., Wurm A.A., Bräuer-Hartmann D., Katzerke C., Hartmann J.-U., Madan V., Müller-Tidow C., Duyster J., Tenen D.G. (2015). NF-κB/STAT5/miR-155 network targets PU.1 in FLT3-ITD-driven acute myeloid leukemia. Leukemia.

[111] Popovic R., Riesbeck L.E., Velu C.S., Chaubey A., Zhang J., Achille N.J., Erfurth F.E., Eaton K., Lu J., Grimes H.L. (2009). Regulation of mir-196b by MLL and its overexpression by MLL fusions contributes to immortalization. Blood.

[112] Li Z., Huang H., Chen P., He M., Li Y., Arnovitz S., Jiang X., He C., Hyjek E., Zhang J. (2012). miR-196b directly targets both HOXA9/MEIS1 oncogenes and FAS tumour suppressor in MLL-rearranged leukaemia. Nat. Commun..

[113] Emmrich S., Katsman-Kuipers J.E., Henke K., Khatib M.E., Jammal R., Engeland F., Dasci F., Zwaan C.M., Boer M.L.D., Verboon L. (2013). miR-9 is a tumor suppressor in pediatric AML with t(8;21). Leukemia.

[114] Senyuk V., Zhang Y., Liu Y., Ming M., Premanand K., Zhou L., Chen P., Chen J., Rowley J.D., Nucifora G. (2013). Critical role of miR-9 in myelopoiesis and EVI1-induced leukemogenesis. Proc. Natl. Acad. Sci. USA.

[115] Sun H., Xie Y., Wu X., Hu W., Chen X., Wu K., Wang H., Zhao S., Shi Q., Wang X. (2024). circRNAs as prognostic markers in pediatric acute myeloid leukemia. Cancer Lett..

[116] Li W., Zhong C., Jiao J., Li P., Cui B., Ji C., Ma D. (2017). Characterization of hsa_circ_0004277 as a New Biomarker for Acute Myeloid Leukemia via Circular RNA Profile and Bioinformatics Analysis. Int. J. Mol. Sci..

[117] Guarnerio J., Bezzi M., Jeong J.C., Paffenholz S.V., Berry K., Naldini M.M., Lo-Coco F., Tay Y., Beck A.H., Pandolfi P.P. (2016). Oncogenic Role of Fusion-circRNAs Derived from Cancer-Associated Chromosomal Translocations. Cell.

[118] Can C., Yang X., Jia H., Wu H., Guo X., Wei Y., Jia Z., Liu W., Zhang A., He N. (2025). Exosomal circ_0006896 promotes AML progression via interaction with HDAC1 and restriction of antitumor immunity. Mol. Cancer.

[119] Zhang X., Weissman S.M., Newburger P.E. (2014). Long intergenic non-coding RNA HOTAIRM1 regulates cell cycle progression during myeloid maturation in NB4 human promyelocytic leukemia cells. RNA Biol..

[120] Zhang X., Lian Z., Padden C., Gerstein M.B., Rozowsky J., Snyder M., Gingeras T.R., Kapranov P., Weissman S.M., Newburger P.E. (2009). A myelopoiesis-associated regulatory intergenic noncoding RNA transcript within the human HOXA cluster. Blood.

[121] Farajzadeh M., Fathi M., Jalali P., Kheshti A.M., Khodayari S., Hojjat-Farsangi M., Jadidi F. (2025). Long noncoding RNAs in acute myeloid leukemia: Biomarkers, prognostic indicators, and treatment potential. Cancer Cell Int..

[122] Maurya S.S., Maurya S., Chaturvedi S.K. (2025). Role of Non-coding RNAs in Acute Myeloid Leukemia. Non-Coding RNA.

[123] Yankova E., Aspris D., Tzelepis K. (2021). The N6-methyladenosine RNA modification in acute myeloid leukemia. Curr. Opin. Hematol..

[124] Hu R., Liao P., Xu B., Qiu Y., Zhang H., Li Y. (2024). N6-methyladenosine RNA modifications: A potential therapeutic target for AML. Ann. Hematol..

[125] Jin Z., MacPherson K., Liu Z., Vu L.P. (2023). RNA modifications in hematological malignancies. Int. J. Hematol..

[126] Barbieri I., Tzelepis K., Pandolfini L., Shi J., Millán-Zambrano G., Robson S.C., Aspris D., Migliori V., Bannister A.J., Han N. (2017). Promoter-bound METTL3 maintains myeloid leukaemia by m6A-dependent translation control. Nature.

[127] Vu L.P., Pickering B.F., Cheng Y., Zaccara S., Nguyen D., Minuesa G., Chou T., Chow A., Saletore Y., Mackay M. (2017). The N6-methyladenosine (m6A)-forming enzyme METTL3 controls myeloid differentiation of normal hematopoietic and leukemia cells. Nat. Med..

[128] Li Z., Weng H., Su R., Weng X., Zuo Z., Li C., Huang H., Nachtergaele S., Dong L., Hu C. (2017). FTO Plays an Oncogenic Role in Acute Myeloid Leukemia as a N6-Methyladenosine RNA Demethylase. Cancer Cell.

[129] Li W., Zhao Y., Wu D., Chen Z., Qing Y., Yang F., Ji F., Zhang L., Sau L., Chen J. (2025). FTO degrader impairs ribosome biogenesis and protein translation in acute myeloid leukemia. Sci. Adv..

[130] Zhang X., Wang X.Q.D., Gore H., Himadewi P., Feng F., Liu J. (2020). 3D Genomics of Acute Meyloid Leukemia Reveals the Imbalance between DNA Methylation Canyon Interactions and Leukemic Specific Enhancer Network Interactions. Blood.

[131] Wang P., Tang Z., Lee B., Zhu J.J., Cai L., Szalaj P., Tian S.Z., Zheng M., Plewczynski D., Ruan X. (2020). Chromatin topology reorganization and transcription repression by PML-RARα in acute promyeloid leukemia. Genome Biol..

[132] Xu J., Song F., Lyu H., Kobayashi M., Zhang B., Zhao Z., Hou Y., Wang X., Luan Y., Jia B. (2022). Subtype-specific 3D genome alteration in acute myeloid leukemia. Nature.

[133] Sui P., Wang Z., Zhang P., Pan F. (2024). Three-dimensional chromatin landscapes in MLLr AML. Exp. Hematol. Oncol..

[134] Fischer A., Hernández-Rodríguez B., Mulet-Lazaro R., Nuetzel M., Hölzl F., van Herk S., Kavelaars F.G., Stanewsky H., Ackermann U., Niang A.H. (2024). STAG2 mutations reshape the cohesin-structured spatial chromatin architecture to drive gene regulation in acute myeloid leukemia. Cell Rep..

[135] Darici S., Alkhaldi H., Horne G., Jørgensen H.G., Marmiroli S., Huang X. (2020). Targeting PI3K/Akt/mTOR in AML: Rationale and Clinical Evidence. J. Clin. Med..

[136] Lo Schiavo F., Salvesi C., Jandoubi M., Pirini F., Garbetta J., Martinelli G., Simonetti G., Ferrari A., Lo Schiavo F., Salvesi C. (2025). Novel molecular mechanisms of FLT3 deregulation: From the acute myeloid leukemia experience to therapeutic insights in acute lymphoblastic leukemia. Mol. Cancer.

[137] Chen W., Drakos E., Grammatikakis I., Schlette E.J., Li J., Leventaki V., Staikou-Drakopoulou E., Patsouris E., Panayiotidis P., Medeiros L.J. (2010). mTOR signaling is activated by FLT3 kinase and promotes survival of FLT3-mutated acute myeloid leukemia cells. Mol. Cancer.

[138] Carter J.L., Hege K., Yang J., Kalpage H.A., Su Y., Edwards H., Hüttemann M., Taub J.W., Ge Y., Carter J.L. (2020). Targeting multiple signaling pathways: The new approach to acute myeloid leukemia therapy. Signal Transduct. Target. Ther..

[139] Sueur G., Boutet A., Gotanègre M., Mas V.M.-D., Besson A., Manenti S., Bertoli S. (2020). STAT5-dependent regulation of CDC25A by miR-16 controls proliferation and differentiation in FLT3-ITD acute myeloid leukemia. Sci. Rep..

[140] Schepers H., van Gosliga D., Wierenga A.T.J., Eggen B.J.L., Schuringa J.J., Vellenga E. (2007). STAT5 is required for long-term maintenance of normal and leukemic human stem/progenitor cells. Blood.

[141] Chen Y., Yin Z., Westover K.D., Zhou Z., Shu L. (2025). Advances and Challenges in RAS Signaling Targeted Therapy in Leukemia. Mol. Cancer Ther..

[142] Kaburagi T., Yamato G., Shiba N., Yoshida K., Hara Y., Tabuchi K., Shiraishi Y., Ohki K., Sotomatsu M., Arakawa H. (2022). Clinical significance of RAS pathway alterations in pediatric acute myeloid leukemia. Haematologica.

[143] Huang X., Schwind S., Santhanam R., Eisfeld A.-K., Chiang C.-l., Lankenau M., Yu B., Hoellerbauer P., Jin Y., Tarighat S.S. (2016). Targeting the RAS/MAPK pathway with miR-181a in acute myeloid leukemia. Oncotarget.

[144] Pomeroy E.J., Eckfeldt C.E. (2017). Targeting Ras signaling in AML: RALB is a small GTPase with big potential. Small GTPases.

[145] Kampen K.R., ter Elst A., de Bont E.S.J.M. (2013). Vascular endothelial growth factor signaling in acute myeloid leukemia. Cell. Mol. Life Sci. CMLS.

[146] Anderson N.R., Sheth V., Li H., Harris M.W., Qiu S., Crossman D.K., Kumar H., Agarwal P., Nagasawa T., Paterson A.J. (2023). Microenvironmental CXCL12 deletion enhances Flt3-ITD acute myeloid leukemia stem cell response to therapy by reducing p38 MAPK signaling. Leukemia.

[147] Koistinen P., Siitonen T., Mäntymaa P., Säily M., Kinnula V., Savolainen E.-R., Soini Y. (2001). Regulation of the acute myeloid leukemia cell line OCI/AML-2 by endothelial nitric oxide synthase under the control of a vascular endothelial growth factor signaling system. Leukemia.

[148] Winkler I.G., Barbier V., Pattabiraman D.R., Gonda T.J., Magnani J.L., Levesque J.-P. (2014). Vascular Niche E-Selectin Protects Acute Myeloid Leukaemia Stem Cells from Chemotherapy. Blood.

[149] Winkler I.G., Barbier V., Tay J., Levesque J.-P., Magnani J.L., Fiveash C.E., Erbani J.D. (2019). Blocking Vascular Niche E-Selectin Dampens AML Stem Cell Regeneration/Survival Potential In Vivo By Inhibiting MAPK/ERK and PI3K/AKT Signalling Pathways. Blood.

[150] Cho B.-S., Kim H.-J., Konopleva M. (2017). Targeting the CXCL12/CXCR4 axis in acute myeloid leukemia: From bench to bedside. Korean J. Intern. Med..

[151] Kojima K., McQueen T., Chen Y., Jacamo R., Konopleva M., Shinojima N., Shpall E., Huang X., Andreeff M. (2011). p53 activation of mesenchymal stromal cells partially abrogates microenvironment-mediated resistance to FLT3 inhibition in AML through HIF-1α-mediated down-regulation of CXCL12. Blood.

[152] Bakhtiyari M., Liaghat M., Aziziyan F., Shapourian H., Yahyazadeh S., Alipour M., Shahveh S., Maleki-Sheikhabadi F., Halimi H., Forghaniesfidvajani R. (2023). The role of bone marrow microenvironment (BMM) cells in acute myeloid leukemia (AML) progression: Immune checkpoints, metabolic checkpoints, and signaling pathways. Cell Commun. Signal..

[153] Urs A.P., Goda C., Kulkarni R. (2024). Remodeling of the bone marrow microenvironment during acute myeloid leukemia progression. Ann. Transl. Med..

[154] Láinez-González D., Alonso-Aguado A.B., Alonso-Dominguez J.M. (2023). Understanding the Wnt Signaling Pathway in Acute Myeloid Leukemia Stem Cells: A Feasible Key against Relapses. Biology.

[155] Cheng C.K., Li L., Cheng S.H., Ng K., Chan N.P.H., Ip R.K.L., Wong R.S.M., Shing M.M.K., Li C.K., Ng M.H.L. (2011). Secreted-frizzled related protein 1 is a transcriptional repression target of the t(8;21) fusion protein in acute myeloid leukemia. Blood.

[156] Valencia A., Román-Gómez J., Cervera J., Such E., Barragán E., Bolufer P., Moscardó F., Sanz G.F., Sanz M.A. (2009). Wnt signaling pathway is epigenetically regulated by methylation of Wnt antagonists in acute myeloid leukemia. Leukemia.

[157] Griffiths E.A., Gore S.D., Hooker C., McDevitt M.A., Karp J.E., Smith B.D., Mohammad H.P., Ye Y., Herman J.G., Carraway H.E. (2010). Acute myeloid leukemia is characterized by Wnt pathway inhibitor promoter hypermethylation. Leuk. Lymphoma.

[158] Wang Y., Krivtsov A.V., Sinha A.U., North T.E., Goessling W., Feng Z., Zon L.I., Armstrong S.A. (2010). The Wnt/beta-catenin pathway is required for the development of leukemia stem cells in AML. Science.

[159] Kannan S., Sutphin R.M., Hall M.G., Golfman L.S., Fang W., Nolo R.M., Akers L.J., Hammitt R.A., McMurray J.S., Kornblau S.M. (2013). Notch activation inhibits AML growth and survival: A potential therapeutic approach. J. Exp. Med..

[160] Kang Y.-A., Pietras E.M., Passegué E. (2019). Deregulated Notch and Wnt signaling activates early-stage myeloid regeneration pathways in leukemia. J. Exp. Med..

[161] Shallis R.M., Bewersdorf J.P., Boddu P.C., Zeidan A.M. (2019). Hedgehog pathway inhibition as a therapeutic target in acute myeloid leukemia. Expert Rev. Anticancer. Ther..

[162] Lau B.W., Huh K., Madero-Marroquin R., De Marchi F., Lim Y., Wang Q., Lobo F., Marchionni L., Smith D.B., DeZern A. (2018). Hedgehog/GLI1 activation leads to leukemic transformation of myelodysplastic syndrome in vivo and GLI1 inhibition results in antitumor activity. Oncogene.

[163] Fukushima N., Minami Y., Kakiuchi S., Kuwatsuka Y., Hayakawa F., Jamieson C., Kiyoi H., Naoe T. (2016). Small-molecule Hedgehog inhibitor attenuates the leukemia-initiation potential of acute myeloid leukemia cells. Cancer Sci..

[164] Siggins S.L., Nguyen N.-Y.N., McCormack M.P., Vasudevan S., Villani R., Jane S.M., Wainwright B.J., Curtis D.J. (2009). The Hedgehog receptor Patched1 regulates myeloid and lymphoid progenitors by distinct cell-extrinsic mechanisms. Blood.

[165] Shimosato Y., Yamamoto K., Jia Y., Zhang W., Shiba N., Hayashi Y., Ito S., Kitamura T., Goyama S. (2025). NPM1-fusion proteins promote myeloid leukemogenesis through XPO1-dependent HOX activation. Leukemia.

[166] Zhou X., Lu R. (2023). HOXA9/MEIS1 targets in leukemia: Reinforced signaling networks and therapeutic opportunities. Haematologica.

[167] Mohr S., Doebele C., Comoglio F., Berg T., Beck J., Bohnenberger H., Alexe G., Corso J., Ströbel P., Wachter A. (2017). Hoxa9 and Meis1 Cooperatively Induce Addiction to Syk Signaling by Suppressing miR-146a in Acute Myeloid Leukemia. Cancer Cell.

[168] Garcia-Cuellar M.-P., Prinz A., Slany R.K. (2022). Meis1 supports leukemogenesis through stimulation of ribosomal biogenesis and Myc. Haematologica.

[169] Fiskus W., Boettcher S., Daver N., Mill C.P., Sasaki K., Birdwell C.E., Davis J.A., Takahashi K., Kadia T.M., DiNardo C.D. (2022). Effective Menin inhibitor-based combinations against AML with MLL rearrangement or NPM1 mutation (NPM1c). Blood Cancer J..

[170] Zawacka J.E. (2024). p53 biology and reactivation for improved therapy in MDS and AML. Biomark. Res..

[171] Lacroix M., Riscal R., Arena G., Linares L.K., Le Cam L. (2020). Metabolic functions of the tumor suppressor p53: Implications in normal physiology, metabolic disorders, and cancer. Mol. Metab..

[172] Quintás-Cardama A., Hu C., Qutub A., Qiu Y.H., Zhang X., Post S.M., Zhang N., Coombes K., Kornblau S.M. (2017). p53 pathway dysfunction is highly prevalent in acute myeloid leukemia independent of TP53 mutational status. Leukemia.

[173] Ghimire B., Zimmer M., Donthireddy V. (2025). TP53-Mutated Acute Myeloid Leukemia: Review of Treatment and Challenges. Eur. J. Haematol..

[174] Shahzad M., Amin M.K., Daver N.G., Shah M.V., Hiwase D., Arber D.A., Kharfan-Dabaja M.A., Badar T., Shahzad M., Amin M.K. (2024). What have we learned about TP53-mutated acute myeloid leukemia?. Blood Cancer J..

[175] Vogler M., Braun Y., Smith V.M., Westhoff M.-A., Pereira R.S., Pieper N.M., Anders M., Callens M., Vervliet T., Abbas M. (2025). The BCL2 family: From apoptosis mechanisms to new advances in targeted therapy. Signal Transduct. Target. Ther..

[176] Nwosu G.O., Ross D.M., Powell J.A., Pitson S.M., Nwosu G.O., Ross D.M., Powell J.A., Pitson S.M. (2024). Venetoclax therapy and emerging resistance mechanisms in acute myeloid leukaemia. Cell Death Dis..

[177] Campos L., Rouault J., Sabido O., Oriol P., Roubi N., Vasselon C., Archimbaud E., Magaud J., Guyotat D. (1993). High expression of bcl-2 protein in acute myeloid leukemia cells is associated with poor response to chemotherapy. Blood.

[178] Wei Y., Cao Y., Sun R., Cheng L., Xiong X., Jin X., He X., Lu W., Zhao M. (2020). Targeting Bcl-2 Proteins in Acute Myeloid Leukemia. Front. Oncol..

[179] Garciaz S., Saillard C., Hicheri Y., Hospital M.-A., Vey N., Garciaz S., Saillard C., Hicheri Y., Hospital M.-A., Vey N. (2021). Venetoclax in Acute Myeloid Leukemia: Molecular Basis, Evidences for Preclinical and Clinical Efficacy and Strategies to Target Resistance. Cancers.

[180] Hagen J.T., Montgomery M.M., Aruleba R.T., Chrest B.R., Krassovskaia P., Green T.D., Pacheco E.A., Kassai M., Zeczycki T.N., Schmidt C.A. (2025). Acute myeloid leukemia mitochondria hydrolyze ATP to support oxidative metabolism and resist chemotherapy. Sci. Adv..

[181] Thomalla D., Beckmann L., Grimm C., Oliverio M., Meder L., Herling C., Nieper P., Feldmann T., Merkel O., Lorsy E. (2022). Deregulation and epigenetic modification of BCL2-family genes cause resistance to venetoclax in hematologic malignancies. Blood.

[182] Thus Y.J., Rooij M.F.d., Swier N., Beijersbergen R.L., Guikema J.E., Kersten M.-J., Eldering E., Pals S.T., Kater A.P., Spaargaren M. (2022). Inhibition of casein kinase 2 sensitizes mantle cell lymphoma to venetoclax through MCL-1 downregulation. Haematologica.

[183] Choi H.-S., Kim B.S., Yoon S., Oh S.-O., Lee D. (2024). Leukemic Stem Cells and Hematological Malignancies. Int. J. Mol. Sci..

[184] Stelmach P., Trumpp A. (2023). Leukemic stem cells and therapy resistance in acute myeloid leukemia. Haematologica.

[185] Zeijlemaker W., Grob T., Meijer R., Hanekamp D., Kelder A., Carbaat-Ham J.C., Oussoren-Brockhoff Y.J.M., Snel A.N., Veldhuizen D., Scholten W.J. (2019). CD34^+^CD38^−^ leukemic stem cell frequency to predict outcome in acute myeloid leukemia. Leukemia.

[186] Yanagisawa B., Perkins B., Karantanos T., Levis M., Ghiaur G., Smith B.D., Jones R.J. (2020). Expression of putative leukemia stem cell targets in genetically-defined acute myeloid leukemia subtypes. Leuk. Res..

[187] Pabst C., Bergeron A., Lavallée V.-P., Yeh J., Gendron P., Norddahl G.L., Krosl J., Boivin I., Deneault E., Simard J. (2016). GPR56 identifies primary human acute myeloid leukemia cells with high repopulating potential in vivo. Blood.

[188] Jin L., Hope K.J., Zhai Q., Smadja-Joffe F., Dick J.E. (2006). Targeting of CD44 eradicates human acute myeloid leukemic stem cells. Nat. Med..

[189] Ehninger A., Kramer M., Röllig C., Thiede C., Bornhäuser M., Von Bonin M., Wermke M., Feldmann A., Bachmann M., Ehninger G. (2014). Distribution and levels of cell surface expression of CD33 and CD123 in acute myeloid leukemia. Blood Cancer J..

[190] Boucher J.C., Shrestha B., Vishwasrao P., Leick M., Cervantes E.V., Ghafoor T., Reid K., Spitler K., Yu B., Betts B.C. (2023). Bispecific CD33/CD123 targeted chimeric antigen receptor T cells for the treatment of acute myeloid leukemia. Mol. Ther. Oncolytics.

[191] Al-Hussaini M., Rettig M.P., Ritchey J.K., Karpova D., Uy G.L., Eissenberg L.G., Gao F., Eades W.C., Bonvini E., Chichili G.R. (2016). Targeting CD123 in acute myeloid leukemia using a T-cell-directed dual-affinity retargeting platform. Blood.

[192] Zeng Z., Roobrouck A., Deschamps G., Bonnevaux H., Guerif S., De Brabandere V., Amara C., Dejonckheere E., Virone-Oddos A., Chiron M. (2024). Dual-targeting CD33/CD123 NANOBODY T-cell engager with potent anti-AML activity and good safety profile. Blood Adv..

[193] Sakoda T., Kikushige Y., Irifune H., Kawano G., Harada T., Semba Y., Hayashi M., Shima T., Mori Y., Eto T. (2024). TIM-3 marks measurable residual leukemic stem cells responsible for relapse after allogeneic stem cell transplantation. Cancer Sci..

[194] Eladl E., Tremblay-LeMay R., Rastgoo N., Musani R., Chen W., Liu A., Chang H. (2020). Role of CD47 in Hematological Malignancies. J. Hematol. Oncol..

[195] Jaiswal S., Jamieson C.H., Pang W.W., Park C.Y., Chao M.P., Majeti R., Traver D., van Rooijen N., Weissman I.L. (2009). CD47 is upregulated on circulating hematopoietic stem cells and leukemia cells to avoid phagocytosis. Cell.

[196] Gudmundsson K.O., Du Y. (2023). Quiescence regulation by normal haematopoietic stem cells and leukaemia stem cells. FEBS J..

[197] Yu H., Yan L. (2025). Mitochondrial metabolism: Critical mechanisms underpinning normal hematopoietic stem cell and acute myeloid leukemia stem cell function. Gene.

[198] Pei S., Minhajuddin M., Adane B., Khan N., Stevens B.M., Mack S.C., Lai S., Rich J.N., Inguva A., Shannon K.M. (2018). AMPK/FIS1-Mediated Mitophagy Is Required for Self-Renewal of Human AML Stem Cells. Cell Stem Cell.

[199] Lagadinou E.D., Sach A., Callahan K., Rossi R.M., Neering S.J., Minhajuddin M., Ashton J.M., Pei S., Grose V., O’Dwyer K.M. (2013). BCL-2 inhibition targets oxidative phosphorylation and selectively eradicates quiescent human leukemia stem cells. Cell Stem Cell.

[200] Ye H., Adane B., Khan N., Sullivan T., Minhajuddin M., Gasparetto M., Stevens B., Pei S., Balys M., Ashton J.M. (2016). Leukemic Stem Cells Evade Chemotherapy by Metabolic Adaptation to an Adipose Tissue Niche. Cell Stem Cell.

[201] Kaufmann K.B., Garcia-Prat L., Liu Q., Ng S.W.K., Takayanagi S.-I., Mitchell A., Wienholds E., van Galen P., Cumbaa C.A., Tsay M.J. (2019). A stemness screen reveals C3orf54/INKA1 as a promoter of human leukemia stem cell latency. Blood.

[202] Sheng Y., Yu C., Liu Y., Hu C., Ma R., Lu X., Ji P., Chen J., Mizukawa B., Huang Y. (2020). FOXM1 regulates leukemia stem cell quiescence and survival in MLL-rearranged AML. Nat. Commun..

[203] Lechman E.R., Gentner B., Ng S.W., Schoof E.M., van Galen P., Kennedy J.A., Nucera S., Ciceri F., Kaufmann K.B., Takayama N. (2016). miR-126 Regulates Distinct Self-Renewal Outcomes in Normal and Malignant Hematopoietic Stem Cells. Cancer Cell.

[204] Yankova E., Blackaby W., Albertella M., Rak J., De Braekeleer E., Tsagkogeorga G., Pilka E.S., Aspris D., Leggate D., Hendrick A.G. (2021). Small-molecule inhibition of METTL3 as a strategy against myeloid leukaemia. Nature.

[205] Weng H., Huang H., Wu H., Qin X., Zhao B.S., Dong L., Shi H., Skibbe J., Shen C., Hu C. (2018). METTL14 Inhibits Hematopoietic Stem/Progenitor Differentiation and Promotes Leukemogenesis via mRNA m6A Modification. Cell Stem Cell.

[206] Han L., Dong L., Leung K., Zhao Z., Li Y., Gao L., Chen Z., Xue J., Qing Y., Li W. (2023). METTL16 drives leukemogenesis and leukemia stem cell self-renewal by reprogramming BCAA metabolism. Cell Stem Cell.

[207] Tabe Y., Konopleva M., Andreeff M. (2020). Fatty Acid Metabolism, Bone Marrow Adipocytes, and AML. Front. Oncol..

[208] Xu Y., Mou J., Wang Y., Zhou W., Rao Q., Xing H., Tian Z., Tang K., Wang M., Wang J. (2022). Regulatory T cells promote the stemness of leukemia stem cells through IL10 cytokine-related signaling pathway. Leukemia.

[209] Miari K.E., Guzman M.L., Wheadon H., Williams M.T.S. (2021). Macrophages in Acute Myeloid Leukaemia: Significant Players in Therapy Resistance and Patient Outcomes. Front. Cell Dev. Biol..

[210] Padró T., Ruiz S., Bieker R., Bürger H., Steins M., Kienast J., Büchner T., Berdel W.E., Mesters R.M. (2000). Increased angiogenesis in the bone marrow of patients with acute myeloid leukemia. Blood.

[211] Fiedler W., Graeven U., Ergün S.L., Verago S., Kilic N., Stockschläder M., Hossfeld D.K. (1997). Vascular Endothelial Growth Factor, a Possible Paracrine Growth Factor in Human Acute Myeloid Leukemia. Blood.

[212] Jacamo R., Chen Y., Wang Z., Ma W., Zhang M., Spaeth E.L., Wang Y., Battula V.L., Mak P.Y., Schallmoser K. (2014). Reciprocal leukemia-stroma VCAM-1/VLA-4-dependent activation of NF-κB mediates chemoresistance. Blood.

[213] Winkler I.G., Barbier V., Nowlan B., Jacobsen R.N., Forristal C.E., Patton J.T., Magnani J.L., Lévesque J.-P., Winkler I.G., Barbier V. (2012). Vascular niche E-selectin regulates hematopoietic stem cell dormancy, self renewal and chemoresistance. Nat. Med..

[214] Barbier V., Erbani J., Fiveash C., Davies J.M., Tay J., Tallack M.R., Lowe J., Magnani J.L., Pattabiraman D.R., Perkins A.C. (2020). Endothelial E-selectin inhibition improves acute myeloid leukaemia therapy by disrupting vascular niche-mediated chemoresistance. Nat. Commun..

[215] Ding L., Saunders T.L., Enikolopov G., Morrison S.J., Ding L., Saunders T.L., Enikolopov G., Morrison S.J. (2012). Endothelial and perivascular cells maintain haematopoietic stem cells. Nature.

[216] Fodil S., Arnaud M., Vaganay C., Puissant A., Lengline E., Mooney N., Itzykson R., Zafrani L. (2022). Endothelial cells: Major players in acute myeloid leukaemia. Blood Rev..

[217] Chen Q., Yuan Y., Chen T. (2014). Morphology, differentiation and adhesion molecule expression changes of bone marrow mesenchymal stem cells from acute myeloid leukemia patients. Mol. Med. Rep..

[218] Battula V.L., Le P.M., Sun J.C., Nguyen K., Yuan B., Zhou X., Sonnylal S., McQueen T., Ruvolo V., Michel K.A. (2017). AML-induced osteogenic differentiation in mesenchymal stromal cells supports leukemia growth. JCI Insight.

[219] Chandran P., Le Y., Li Y., Sabloff M., Mehic J., Rosu-Myles M., Allan D.S. (2015). Mesenchymal stromal cells from patients with acute myeloid leukemia have altered capacity to expand differentiated hematopoietic progenitors. Leuk. Res..

[220] Guardia R.D.d.l., Lopez-Millan B., Lavoie J.R., Bueno C., Castaño J., Gómez-Casares M., Vives S., Palomo L., Juan M., Delgado J. (2017). Detailed Characterization of Mesenchymal Stem/Stromal Cells from a Large Cohort of AML Patients Demonstrates a Definitive Link to Treatment Outcomes. Stem Cell Rep..

[221] Schroeder T., Geyh S., Germing U., Haas R. (2016). Mesenchymal stromal cells in myeloid malignancies. Blood Res..

[222] Hou D., Cai D., Dai W., Liao X., Tan M., Zheng X., Wang L., Liu J., Wang J., Wang X. (2025). Targeting bone marrow mesenchymal stromal cell-derived IL-6 to overcome acute myeloid leukemia chemoresistance. Blood Adv..

[223] Forte D., García-Fernández M., Sánchez-Aguilera A., Stavropoulou V., Fielding C., Martín-Pérez D., López J.A., Costa A.S., Tronci L., Nikitopoulou E. (2020). Bone Marrow Mesenchymal Stem Cells Support Acute Myeloid Leukemia Bioenergetics and Enhance Antioxidant Defense and Escape from Chemotherapy. Cell Metab..

[224] Lisi-Vega L.E., Pievani A., García-Fernández M., Forte D., Williams T.L., Serafini M., Méndez-Ferrer S. (2025). Bone marrow mesenchymal stromal cells support translation in refractory acute myeloid leukemia. Cell Rep..

[225] Wang W., Bochtler T., Wuchter P., Manta L., He H., Eckstein V., Ho A.D., Lutz C. (2017). Mesenchymal stromal cells contribute to quiescence of therapy-resistant leukemic cells in acute myeloid leukemia. Eur. J. Haematol..

[226] Li Y., Zhang C., Zhang Y., Huang Y., Yuan X., Yang B., He Z., Liu Y., Wang F. (2025). Acute myeloid leukemia cells induce senescence and adipogenic differentiation of mesenchymal stem cells in a tumor-supportive microenvironment at the same time. Cell. Signal..

[227] Ahmed H., Patnana P.K., Al-Matary Y.S., Fiori M., Vorwerk J., Ahmad M.H., Dazert E., Oelschläger L., Künstner A., Opalka B. (2025). Impact of Acute Myeloid Leukemia Cells on the Metabolic Function of Bone Marrow Mesenchymal Stem Cells. Int. J. Mol. Sci..

[228] Saadallah K., Vianay B., Bonnemay L., Pasquer H., Kelly L., Mathis S., Culeux C., Marie R., Meslin P.A., Fodil S. (2025). AML patient blasts exhibit polarization defects upon interaction with bone marrow stromal cells. EMBO Rep..

[229] Liu M., Yang M., Qi Y., Ma Y., Guo Q., Guo L., Liu C., Liu W., Xiao L., Yang Y. (2025). Immunosuppressive cells in acute myeloid leukemia: Mechanisms and therapeutic target. Front. Immunol..

[230] Taghiloo S., Asgarian-Omran H. (2021). Immune evasion mechanisms in acute myeloid leukemia: A focus on immune checkpoint pathways. Crit. Rev. Oncol. Hematol..

[231] Wang Z., Liu T., Li Y., Li Z., Bi K. (2024). Increased Th17 and Treg levels in peripheral blood positively correlate with minimal residual disease in acute myeloid leukaemia. Hematology.

[232] Swatler J., Turos-Korgul L., Brewinska-Olchowik M., De Biasi S., Dudka W., Le B.V., Kominek A., Cyranowski S., Pilanc P., Mohammadi E. (2022). 4-1BBL-containing leukemic extracellular vesicles promote immunosuppressive effector regulatory T cells. Blood Adv..

[233] Agha D.M., Rouas R., Najar M., Bouhtit F., Fayyad-Kazan H., Lagneaux L., Bron D., Meuleman N., Lewalle P., Merimi M. (2020). Impact of Bone Marrow miR-21 Expression on Acute Myeloid Leukemia T Lymphocyte Fragility and Dysfunction. Cells.

[234] Francisco L.M., Salinas V.H., Brown K.E., Vanguri V.K., Freeman G.J., Kuchroo V.K., Sharpe A.H. (2009). PD-L1 regulates the development, maintenance, and function of induced regulatory T cells. J. Exp. Med..

[235] Zhou Q., Munger M.E., Highfill S.L., Tolar J., Weigel B.J., Riddle M., Sharpe A.H., Vallera D.A., Azuma M., Levine B.L. (2010). Program death-1 signaling and regulatory T cells collaborate to resist the function of adoptively transferred cytotoxic T lymphocytes in advanced acute myeloid leukemia. Blood.

[236] Dong Y., Han Y., Huang Y., Jiang S., Huang Z., Chen R., Yu Z., Yu K., Zhang S. (2020). PD-L1 Is Expressed and Promotes the Expansion of Regulatory T Cells in Acute Myeloid Leukemia. Front. Immunol..

[237] Wells G., Kennedy P.T., Dahal L.N. (2021). Investigating the Role of Indoleamine 2,3-Dioxygenase in Acute Myeloid Leukemia: A Systematic Review. Front. Immunol..

[238] Hakak R., Poopak B., Majd A. (2024). Increased IDO expression and regulatory T cells in acute myeloid leukemia: Implications for immune escape and therapeutic targeting. Blood Res..

[239] Mansour I., Zayed R.A., Said F., Latif L.A. (2016). Indoleamine 2,3-dioxygenase and regulatory T cells in acute myeloid leukemia. Hematology.

[240] Arandi N., Ramzi M., Safaei F., Monabati A. (2018). Overexpression of indoleamine 2,3-dioxygenase correlates with regulatory T cell phenotype in acute myeloid leukemia patients with normal karyotype. Blood Res..

[241] Mellor A.L., Munn D.H. (2004). IDO expression by dendritic cells: Tolerance and tryptophan catabolism. Nat. Rev. Immunol..

[242] Yamaguchi T., Kishi A., Osaki M., Morikawa H., Prieto-Martin P., Wing K., Saito T., Sakaguchi S. (2013). Construction of self-recognizing regulatory T cells from conventional T cells by controlling CTLA-4 and IL-2 expression. Proc. Natl. Acad. Sci. USA.

[243] Overacre-Delgoffe A.E., Chikina M., Dadey R.E., Yano H., Brunazzi E.A., Shayan G., Horne W., Moskovitz J.M., Kolls J.K., Sander C. (2017). Interferon-γ Drives Treg Fragility to Promote Anti-tumor Immunity. Cell.

[244] Wu H., Li P., Shao N., Ma J., Ji M., Sun X., Ma D., Ji C. (2012). Aberrant expression of Treg-associated cytokine IL-35 along with IL-10 and TGF-β in acute myeloid leukemia. Oncol. Lett..

[245] Collison L.W., Workman C.J., Kuo T.T., Boyd K., Wang Y., Vignali K.M., Cross R., Sehy D., Blumberg R.S., Vignali D.A.A. (2007). The inhibitory cytokine IL-35 contributes to regulatory T-cell function. Nature.

[246] Cao X., Cai S.F., Fehniger T.A., Song J., Collins L.I., Piwnica-Worms D.R., Ley T.J. (2007). Granzyme B and perforin are important for regulatory T cell-mediated suppression of tumor clearance. Immunity.

[247] Xu Z.-J., Gu Y., Wang C.-Z., Jin Y., Wen X.-M., Ma J.-C., Tang L.-J., Mao Z.-W., Qian J., Lin J. (2019). The M2 macrophage marker CD206: A novel prognostic indicator for acute myeloid leukemia. Oncoimmunology.

[248] Al-Matari H.A., Mansour A.B., Ibrahim S.A., Zaki N.E.-S., Badr A.M., Al-Matari H.A., Mansour A.B., Ibrahim S.A., Zaki N.E.-S., Badr A.M. (2024). Soluble CD206 and CD163 as novel prognostic biomarkers in adult acute myeloid leukemia. J. Basic Appl. Zool..

[249] Smirnova T., Spertini C., Spertini O. (2021). CSF1R Inhibition Combined with GM-CSF Reprograms Macrophages and Disrupts Protumoral Interplays with AML Cells. Cancers.

[250] Zhang D., Cui X., Li Y., Wang R., Wang H., Dai Y., Ren Q., Wang L., Zheng G. (2023). Sox13 and M2-like leukemia-associated macrophages contribute to endogenous IL-34 caused accelerated progression of acute myeloid leukemia. Cell Death Dis..

[251] Spertini C., Bénéchet A.P., Birch F., Bellotti A., Román-Trufero M., Arber C., Auner H.W., Mitchell R.A., Spertini O., Smirnova T. (2024). Macrophage migration inhibitory factor blockade reprograms macrophages and disrupts prosurvival signaling in acute myeloid leukemia. Cell Death Discov..

[252] Al-Matary Y.S., Botezatu L., Opalka B., Hönes J.M., Lams R.F., Thivakaran A., Schütte J., Köster R., Lennartz K., Schroeder T. (2016). Acute myeloid leukemia cells polarize macrophages towards a leukemia supporting state in a Growth factor independence 1 dependent manner. Haematologica.

[253] Tian C., Li Y., Si J., Kang J., Chen Z., Nuermaimaiti R., Wang Y., Yu Y., Zhao Z., Wang X. (2023). Knockdown of let-7b in leukemia associated macrophages inhibit acute myeloid leukemia progression. Hematol. Oncol..

[254] Weinhäuser I., Pereira-Martins D.A., Almeida L.Y., Hilberink J.R., Silveira D.R.A., Quek L., Ortiz C., Araujo C.L., Bianco T.M., Lucena-Araujo A. (2023). M2 macrophages drive leukemic transformation by imposing resistance to phagocytosis and improving mitochondrial metabolism. Sci. Adv..

[255] Jha A.K., Huang S.C.-C., Sergushichev A., Lampropoulou V., Ivanova Y., Loginicheva E., Chmielewski K., Stewart K.M., Ashall J., Everts B. (2015). Network Integration of Parallel Metabolic and Transcriptional Data Reveals Metabolic Modules that Regulate Macrophage Polarization. Immunity.

[256] Zeng W., Li F., Jin S., Ho P.-C., Liu P.-S., Xie X., Zeng W., Li F., Jin S., Ho P.-C. (2023). Functional polarization of tumor-associated macrophages dictated by metabolic reprogramming. J. Exp. Clin. Cancer Res..

[257] Wang R., Zhuang J., Zhang Q., Wu W., Yu X., Zhang H., Xie Z., Wang R., Zhuang J., Zhang Q. (2025). Decoding the metabolic dialogue in the tumor microenvironment: From immune suppression to precision cancer therapies. Exp. Hematol. Oncol..

[258] Feng J., Gou J., Wang Y., Wei W., Ma Y., Ren X., Zhao C., Cheng X., Lei L., Tan Z. (2025). Bisecting GlcNAc expression by bone marrow stromal cells modulates TGF-β1-driven macrophage polarization in myeloid leukemias. Haematologica.

[259] Li C., Xu X., Wei S., Jiang P., Xue L., Wang J. (2021). Tumor-associated macrophages: Potential therapeutic strategies and future prospects in cancer. J. Immunother. Cancer.

[260] Chao M.P., Takimoto C.H., Feng D.D., McKenna K., Gip P., Liu J., Volkmer J.-P., Weissman I.L., Majeti R. (2020). Therapeutic Targeting of the Macrophage Immune Checkpoint CD47 in Myeloid Malignancies. Front. Oncol..

[261] Brauneck F., Fischer B., Witt M., Muschhammer J., Oelrich J., Avelar P.H.d.C., Tsoka S., Bullinger L., Seubert E., Smit D.J. (2022). TIGIT blockade repolarizes AML-associated TIGIT^+^ M2 macrophages to an M1 phenotype and increases CD47-mediated phagocytosis. J. Immunother. Cancer.

[262] Samarkhazan H.S. (2025). Integrating multi-omics approaches in acute myeloid leukemia (AML): Advancements and clinical implications. Clin. Exp. Med..

[263] Zeng A.G., Iacobucci I., Shah S., Mitchell A., Wong G., Bansal S., Chen D., Gao Q., Kim H., Kennedy J.A. (2025). Single-cell Transcriptional Atlas of Human Hematopoiesis Reveals Genetic and Hierarchy-Based Determinants of Aberrant AML Differentiation. Blood Cancer Discov..

[264] Reville P.K., Wang B., Marvin-Peek J., Yuan B., Kuo Y.-A., Garza A., Root J., Qiao W., Arruda A., Veletic I. (2025). Blood-based proteomic profiling identifies OSMR as a novel biomarker of AML outcomes. Blood.

[265] Huang F.Y., Trumpp A., Stelmach P. (2025). Resolving leukemic stem cell heterogeneity and plasticity with single-cell multiomics. Semin. Hematol..

[266] Leppä A.-M., Grimes K., Jeong H., Huang F.Y., Andrades A., Waclawiczek A., Boch T., Jauch A., Renders S., Stelmach P. (2024). Single-cell multiomics analysis reveals dynamic clonal evolution and targetable phenotypes in acute myeloid leukemia with complex karyotype. Nat. Genet..

[267] Argüello R.J., Combes A.J., Char R., Gigan J.-P., Baaziz A.I., Bousiquot E., Camosseto V., Samad B., Tsui J., Yan P. (2020). SCENITH: A Flow Cytometry-Based Method to Functionally Profile Energy Metabolism with Single-Cell Resolution. Cell Metab..

[268] Forte D., Pellegrino R.M., Falvo P., Garcia-Gonzalez P., Alabed H.B.R., Maltoni F., Lombardi D., Bruno S., Barone M., Pasini F. (2024). Parallel single-cell metabolic analysis and extracellular vesicle profiling reveal vulnerabilities with prognostic significance in acute myeloid leukemia. Nat. Commun..

[269] Dasdemir E., Veletic I., Ly C.P., Quesada A.E., Jelloul F.Z., Borges P., Basu S., Jindal S., Wang Z., Lazar A.J. (2024). Multimodal Spatial Transcriptomic Profiling Elucidates Niche-Specific Dynamics in Medullary and Extramedullary Acute Myeloid Leukemia. Blood.

[270] Gui G., Bingham M.A., Herzog J.R., Wong-Rolle A., Dillon L.W., Goswami M., Martin E., Reeves J., Kim S., Bahrami A. (2025). Single-cell spatial transcriptomics reveals immunotherapy-driven bone marrow niche remodeling in AML. Sci. Adv..

[271] Mercier F.E., Shi J., Sykes D.B., Oki T., Jankovic M., Man C.H., Kfoury Y.S., Miller E., He S., Zhu A. (2022). In vivo genome-wide CRISPR screening in murine acute myeloid leukemia uncovers microenvironmental dependencies. Blood Adv..

[272] Wang E., Zhou H., Nadorp B., Cayanan G., Chen X., Yeaton A.H., Nomikou S., Witkowski M.T., Narang S., Kloetgen A. (2021). Surface antigen-guided CRISPR screens identify regulators of myeloid leukemia differentiation. Cell Stem Cell.

[273] Nguyen N.H.K., Rafiee R., Parcha P.K., Tagmount A., Rubnitz J., Ribeiro R., Cao X., Pounds S.B., Vulpe C.D., Lamba J.K. (2025). Genome-wide CRISPR/Cas9 screen identifies AraC-daunorubicin-etoposide response modulators associated with outcomes in pediatric AML. Blood Adv..

[274] Pirouzbakht M., Hamzeh S., Soleimani Samarkhazan H., Pirouzbakht M., Hamzeh S., Soleimani Samarkhazan H. (2025). Beyond single biomarkers: Multi-omics strategies to predict immunotherapy outcomes in blood cancers. Clin. Exp. Med..

[275] Haferlach T., Eckardt J.-N., Walter W., Maschek S., Kather J.N., Pohlkamp C., Middeke J.M. (2025). AML diagnostics in the 21st century: Use of AI. Semin. Hematol..

[276] Zhang G., Song C., Yin M., Liu L., Zhang Y., Li Y., Zhang J., Guo M., Li C. (2025). TRAPT: A multi-stage fused deep learning framework for predicting transcriptional regulators based on large-scale epigenomic data. Nat. Commun..

[277] Wang H., Arulraj T., Ippolito A., Popel A.S. (2024). From virtual patients to digital twins in immuno-oncology: Lessons learned from mechanistic quantitative systems pharmacology modeling. NPJ Digit. Med..

[278] Wang Y., Fu T., Xu Y., Ma Z., Xu H., Du B., Lu Y., Gao H., Wu J., Chen J. (2023). TWIN-GPT: Digital Twins for Clinical Trials via Large Language Model. ACM Trans. Multimed. Comput. Commun. Appl..

